# POU5F1 bridges Hedgehog signaling and epithelial remodeling in COPD

**DOI:** 10.3389/fcell.2025.1566251

**Published:** 2025-07-02

**Authors:** Laure M. G. Petit, Randa Belgacemi, Pauline Mulette, Audrey Brisebarre, Lynda Saber Cherif, Maëva A. Devilliers, Sarah Hatoum, Julien Ancel, Gonzague Delepine, Anne Durlach, Myriam Polette, Gaëtan Deslée, Jeanne-Marie Perotin, Valérian Dormoy

**Affiliations:** ^1^ Université de Reims Champagne-Ardenne, INSERM, P3Cell UMR-S1250, Reims, France; ^2^ Lundquist Institute for Biomedical Innovation, Harbor-UCLA Medical Center, Torrance, CA, United States; ^3^ Université de Reims Champagne-Ardenne, INSERM, CHU de Reims, P3Cell UMR-S1250, Reims, France

**Keywords:** Hedgehog, COPD, airways, epithelial cells, Pou5f1

## Abstract

Airway epithelium remodeling is a hallmark of chronic obstructive pulmonary disease (COPD) pathogenesis. Hedgehog signaling is activated during airway epithelial repair to warrant proliferation and during cell differentiation to establish a fully functional epithelium with optimal mucociliary clearance. Consequently, it was found to be altered in COPD patients. Using transcriptomic analysis on air–liquid interface airway epithelial cells during differentiation upon Hedgehog pathway inhibition, we highlighted potential regulators of COPD-associated epithelial remodeling. Furthermore, the alteration of POU5F1 (OCT3/4) was validated in COPD airway epithelial cells and lung tissues. Although further investigations are required, these findings uncovered essential clues tethering respiratory epithelial cell plasticity and Hedgehog signaling.

## 1 Introduction

Epithelial remodeling is a hallmark of chronic obstructive pulmonary disease (COPD) observed in lung tissues as a result of the dysfunction of airway epithelial cell differentiation and inflammatory challenges (Raby et al., 2023; Gohy et al., 2015; Gao et al., 2015). Genomics and transcriptomics have highlighted the association of Hedgehog (HH) effectors with epithelial cell plasticity, along with the declining lung function in COPD patients (Guo et al., 2024; Zhou et al., 2012; Hobbs and Hersh, 2014).

We originally demonstrated that proper airway epithelial cell differentiation relies on HH pathway activation, and the core elements, including GLI2 and SHH, are downregulated in COPD patients (Belgacemi et al., 2020; Ancel et al., 2020). Remodeling of the epithelium is particularly associated with alterations in ciliated cells (Petit et al., 2023; Belgacemi et al., 2021; Perotin et al., 2021). In addition, lung progenitor cells and branching morphogenesis are disrupted during organogenesis in the absence of SHH (Belgacemi et al., 2022). The complex machinery responsible for the fine-tuning of the HH pathway during differentiation has not been elucidated in respiratory research.

In this study, we hypothesized that the HH pathway activation interacts with differentiation markers to maintain airway homeostasis. Therefore, we investigated the key molecular players impacted by HH signaling inhibition during airway epithelial cell differentiation and their potential alterations in COPD patients.

## 2 Materials and methods

### 2.1 Human subjects

COPD patients and non-COPD individuals treated by lung resection or scheduled to undergo fiberoptic bronchoscopy with bronchial brushings (Tables 1, 2) were recruited following standards that were established and approved by the Institutional Review Board of the University Hospital of Reims, France (IRB Reims-CHU 20110612), and included in the cohort for Research and Innovation in Chronic Inflammatory Respiratory Disease (RINNOPARI, NCT02924818). The study included patients with or without COPD who gave their consent. Exclusion criteria included all other acute or chronic respiratory diseases (asthma, cystic fibrosis, bronchiectasis, and pulmonary fibrosis). At enrollment, age, sex, smoking history, and pulmonary function test results were recorded. COPD was defined by post-bronchodilator FEV_1_ (forced expiratory volume in 1 s)/FVC (forced vital capacity) <0.7. Former smokers were considered if their smoking cessation was longer than 1 year.

**TABLE 1:** Clinical characteristics of the patients included in FFPE immunostaining from lung resections and RT-qPCR from isolated AECs.

Bronchial brushing			
Parameters	Non-COPD	COPD	p-value
Number of subjects	6	6	
Gender F/M	1/5	0/6	ns
Age, years	57.5 ± 14.7 [37–76]	67.5 ± 8.9 [60–82]	ns
Smoking history ns			
Never	0	0	
Former	3	2	
Current	3	4	
Pack-years	30 ± 15.8 [10–50]	59.2 ± 15.5 [42–80]	**
Lung functional parameters			
FEV_1_% pred	85.3 ± 11.6 [72–109]	49 ± 22.4 [27–87]	**
FEV_1_/FVC	0.79 ± 0.04 [0.72–0.83]	0.53 ± 0.14 [0.37–0.69]	**

FFPE tissues			
Parameters	Non-COPD	COPD	p-value
Number of subjects	6	8	
Gender F/M	2/4	2/6	ns
Age, years	64.2 ± 5.8 [60–73]	64.3 ± 10.5 [50–81]	ns
Smoking history ns			
Never	1	0	
Former	3	4	
Current	2	4	
Pack-years	45.4 ± 31.4 [7–90]	49.7 ± 19.3 [25–80]	ns
Lung functional parameters			
FEV_1_% pred	110.3 ± 12.1 [94–121]	70.3 ± 13.8 [59–102]	***
FEV_1_/FVC	0.79 ± 0.01 [0.77–0.81]	0.61 ± 0.06 [0.53–0.69]	***

Data are expressed as mean ± SD [min-max], number, or percentage. FEV_1_: Forced expiratory volume in 1 s; FVC: Forced vital capacity. ns: non-significant; **p < 0.01; ***p < 0.001.

**TABLE 2:** Clinical characteristics of the individuals included for RNAseq analysis upon AB5E1 treatment.

Parameters	Non-COPD
Number of subjects	5
Gender F/M	3/2
Age, years	57.2 ± 7.16 [49–67]
Smoking history	
Never	1
Former	2
Current	2
Pack-years	20.8 ± 15.45 [0–42]
Lung functional parameters	
FEV_1_% pred	101.4 ± 21.45 [82–131]
FEV_1_/FVC	0.81 ± 0.05 [0.77–0.86]

Data are expressed as mean ± SD [min–max], number, or percentage. FEV_1_: Forced expiratory volume in 1 s; FVC: Forced vital capacity.

### 2.2 Human primary airway epithelial cell cultures

Isolated airway epithelial cells (AECs) were obtained from bronchial brushings of non-COPD individuals (n = 11) and COPD patients (n = 6) to establish air–liquid interface (ALI) cultures. Six non-COPD individuals and six COPD patients were included to isolate the AECs for PCR analysis (Table 1), and five non-COPD individuals were included to isolate the AECs for RNA sequencing (Table 2), as previously described (Saber Cherif et al., 2022). The cells were recovered by scraping the brushes and dissociation using trypsin-versene. They were counted with ADAM (NanoEnTek, Seoul, South Korea) according to NanoEnTek instructions. Aliquots of 150,000 cells were seeded on 12-well plates containing 0.4-µm Transwells (Corning, Fisher Scientific, New York, NY, United States), which were coated with 0.2 mg/mL collagen type IV from the human placenta (Sigma-Aldrich, Saint-Louis, MO, United States). CnT-17 medium (CellnTec, Bern, Switzerland) was used for initial proliferation in the apical and basal chambers. Upon reaching cell confluency, the apical medium was removed, and PneumaCult-ALI (PnC-ALI, StemCell, Vancouver, BC, Canada) medium was used in the basal chamber. The culture medium was changed three times a week, and the cells were kept up to 35 days in incubators at 37°C, 5% CO_2_. Inhibition of SHH signaling was achieved by AB5E1 (AB_2188307, Interchim, Montluçon, France, 1 μg/mL, diluted in sterile water) addition to the culture medium, as previously shown (Belgacemi et al., 2020).

### 2.3 RNA sequencing

Total RNA from AECs (Table 2) was isolated using a High Pure RNA isolation kit (Roche Diagnostics, Rotkreuz, Switzerland). Libraries were prepared with the NEBNext Ultra II Directional RNA Library Prep Kit for Illumina protocol according to the supplier's recommendations. Briefly, the purification of PolyA-containing mRNA molecules was performed with poly-T oligo-attached magnetic beads from 1 µg of total RNA with the Magnetic mRNA Isolation Kit from NEB (New England Biolabs, Ipswich, MA, United States), and then a fragmentation using divalent cations at an elevated temperature was realized to obtain approximately 300-bp pieces, double-stranded cDNA synthesis. Finally, Illumina adapter ligation and cDNA library amplification were achieved by PCR for sequencing. Sequencing was then carried out on paired-end 100-bp reads on an Illumina NovaSeq. Image analysis and base calling were performed using Illumina Real Time Analysis (version 3.4.4) with the default parameters. Quality control of the raw sequence data was performed using FastQC (version 0.11.5). Head bases were trimmed for adapter sequences, and low-complexity or low-quality sequences were removed with Trimmomatic (version 0.39). The remaining sequences were mapped to the *Homo sapiens* hg38 reference genome assembly (hg38.fa) using tophat2 (version 2.1.1) with stringent parameters, generating BAM format. The quality of alignment was checked using metrics provided by Qualimap (version 2.2.1), and low-quality alignments were removed. Raw counts were obtained using htseq-count (version 0.6.1), normalized using a scaling factor based on median gene expression across the samples, and filtered to exclude genes with fewer than five counts across the samples. The dataset comprising the 10 biosamples was deposited in the GEO with the accession number GSE262404. Gene expressions of *POU5F1* were also collected from datasets publicly available online (GEO database: GSE137557). COPD patients and non-COPD individuals were included, with their clinical features listed in Supplementary Table S1.

The analysis was performed on all genes, and the differences between the two groups were determined using a Student’s t-test with the Benjamini–Hochberg FDR correction. The results are shown on a volcano plot, with 2-fold upregulated genes in red and 2-fold downregulated genes in blue. The data are also shown on a heatmap generated by RStudio.

### 2.4 RT-qPCR analyses

Total RNA from AECs (ALI cultures from bronchial brushings) was isolated by a GenElute RNA Plus Purification Kit (RDP300, Sigma-Aldrich, Saint-Louis, MO, United States), and 250 ng of RNA was reverse-transcribed into cDNA by using a Maxima H Minus cDNA Synthesis Master Kit (MAN0016392, Thermo Scientific, Waltham, MA, United States). Quantitative PCRs were performed with a Luminaris Probe qPCR Master Kit (K0954, ThermoFisher, Waltham, MA, United States) and a TaqMan Gene Expression Assay on a QuantStudio3 instrument (QS3, Thermo Scientific, Waltham, MA, United States) as recommended by the manufacturer. The primers for *POU5F1* were purchased from Thermo Fisher Scientific (Hs00999632_g1), and the primers for *GLI2* and *FOXJ1* were purchased from Eurogentec (Liège, Belgium). The results for the transcript expressions were normalized to the expression of the housekeeping gene *GAPDH* (Hs02758991_g1) and expressed as a fold change (COPD vs. non-COPD) according to the 2^−ΔΔCT^ method.

### 2.5 Single-cell sequencing dataset

The published single-cell sequencing (scRNAseq) data can be found on the Cell×Gene interface under the “Sikkema et al.” dataset (Sikkema et al., 2023). Only lung samples (lung and lung parenchyma) from adults (>18 years old) without respiratory disease (n = 181) were considered. Four categories were defined as regrouping cell populations as established by the Human Lung Cell Atlas (HLCA) consortium. The categories were identified as (1) tracheobronchial epithelial cells (40,822 cells): ciliated columnar cell of the tracheobronchial tree (25,114 cells), club cell (1,816 cells) and respiratory basal cell (13,892 cells); (2) pneumocytes (123,456 cells): type I pneumocyte (19,418 cells) and type II pneumocyte (104,038 cells); (3) stromal cells (120,988 cells): alveolar type 1 fibroblast cell (21,389 cells), alveolar type 2 fibroblast cell (16,752 cells), bronchus fibroblast of lung (921 cells), capillary endothelial cell (37,538 cells), and endothelial cell of lymphatic vessel (8,675 cells), lung pericyte (5,157 cells), myofibroblast cell (1,048 cells), pulmonary artery endothelial cell (10,173 cells), tracheobronchial smooth muscle cell (5,222 cells) and vein endothelial cell (14,113 cells); (4) immune cells (408,371 cells): B cell (6,723 cells), CD1c-positive myeloid dendritic cell (10,628 cells), CD4-positive alpha-beta T cell (35,984 cells), CD8-positive alpha-beta T cell (43,839 cells), alveolar macrophage (174,329 cells), classical monocyte (33,862 cells), elicited macrophage (39,642 cells), lung macrophage (6,331 cells), mast cell (11,712 cells), natural killer cell (30,755 cells), and non-classical monocyte (14,566). The median expression levels and the proportion of expressing cells for HH pathway markers are shown in a bubble plot generated by RStudio with the ggplot2 package. The UMAP was generated with the Cell×Gene interface.

### 2.6 Immunofluorescent staining and analyses

Immunofluorescent staining processes were performed on formalin-fixed paraffin-embedded (FFPE) lung tissue samples distant from the tumor. Three-micrometer FFPE lung tissue section slides were deparaffinized and blocked with 10% BSA in PBS for 30 min at room temperature. The tissue sections were then incubated with the primary antibody (POU5F1: AF1759 R&D Systems, Minneapolis, MN, United States 1:50) for 1 night at 4°C in 3% BSA in PBS. After washing with PBS, a second primary antibody was used for 1 h at room temperature (KP: NBP2-29429 Novus Biologicals, Littleton, CO, United States 1:250). The sections were washed with PBS and incubated with the appropriate secondary antibody in 3% BSA in PBS for 30 min at room temperature. The DNA was stained with DAPI during incubation with the secondary antibody. Micrographs were acquired by an AxioImager Zeiss microscope (20× Ph) with ZEN software (8.1, 2012) and processed with ImageJ (National Institutes of Health) for analysis. For each patient, five random fields per section containing bronchi were taken to evaluate the localization of POU5F1 on epithelial cells. For each field, a threshold was established by subtracting the background and setting the minimum at 0. POU5F1 expression was determined by the pixel mean gray values (PMGVs) in the region of interest, which was defined by the epithelium, in the two groups.

As previously characterized in our study on AECs isolated from nasal polyps (Belgacemi et al., 2020), global remodeling of the epithelium was confirmed based on the ciliated surface of the epithelium at ALI14 with ARL13B immunostaining. Briefly, methanol-fixed AECs from ALI cultures were rehydrated by decreasing the methanol concentration (75%, 50%, and 25%) before a post-fixation in acetone. The cells were blocked with 10% BSA in PBT (PBS +1% DMSO +0.1% Triton) for 2 h at room temperature. The cells were then incubated with an ARL13B (17711-1- AP, ProteinTech, Manchester, United Kingdom, 1:200) for 1 night at 4°C in 3% BSA-PBT. After washing with PBT, the cells were incubated with the appropriate secondary antibody in PBT for 2 h at room temperature. DNA was stained with DAPI during incubation for 15 min. Clarification of cells was achieved by a glycerol gradient (25%, 50%, and 75%) before mounting the slides. Micrographs were acquired by AxioImager Zeiss (20× Ph) with ZEN software (8.1, 2012).

### 2.7 GO terms analysis

Gene ontology (GO) analysis was performed by String on 371 DEGs (differentially expressed genes) (log2FC = −0.5 < DEG < log2FC = 0.5) with three categories: biological process (in green), cellular component (in red), and annotated keywords (in blue). All the statistically significant terms are represented on the histogram.

### 2.8 Statistics

The data are expressed as mean values ± standard deviation (SD). The differences between the two groups were determined using the Student’s t-test (two-tailed) for parametric data and the Mann–Whitney test for non-parametric data. The correlations were analyzed using the Pearson test. A p-value <0.05 was considered significant; *, p < 0.05; **, p < 0.01; ***p < 0.001.

## 3 Results

**FIGURE 1:**
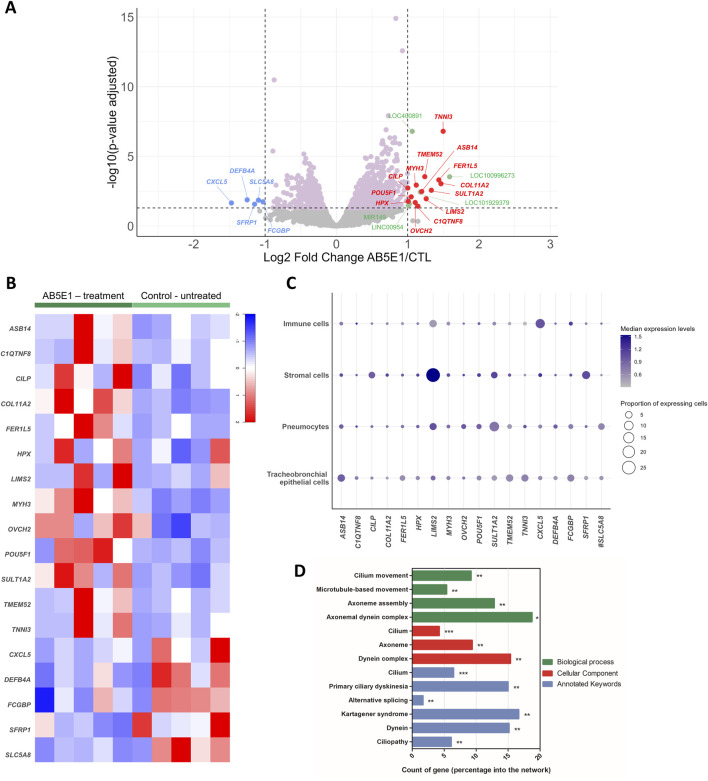
Transcriptomic profiling of AB5E1-treated AECs. **(A)** Volcano plot showing the gene distribution with the p-value adjusted on the y-axis and log2 of the fold change between AB5E1-treated and untreated cells on the x-axis. DEGs are color-coded: pink, log2FC = −0.5 < DEG < log2FC = 0.5; blue, downregulated genes with log2FC < −1; red, upregulated genes with log2FC > 1; green, DEGs corresponding to LOC, LINC, or miRNA with log2FC > 1. **(B)** Heatmap of the 18 most identified DEGs in the AB5E1-treated cell transcriptomes. **(C)** Bubble plot representing cell populations on the y-axis and genes on the x-axis. The size of the dots represents the proportion of expressing cells, and the color intensity represents the median of expression levels. The data were extracted from the full HLCA dataset [except for #, for which data were extracted from the partial HLCA dataset (Sikkema et al., 2023)]. **(D)** Histogram representing a count of genes in GO terms (biological process, cellular component, annotated keywords) for the 371 DEGs (log2FC = −0.5 < DEG < log2FC = 0.5). *p < 0.05; **p < 0.01; ***p < 0.001.

Because airway epithelial cell differentiation is readily analyzable in air–liquid interface cultures and we previously highlighted the involvement of the HH pathway in differentiation with an experimental strategy relying on an antibody targeting SHH (AB5E1), we investigated the global differential transcriptomic signature of the AB5E1-treated cells in the early steps of the differentiation process (ALI7) in AECs isolated from non-COPD individuals with varying smoking histories (Table 2) (Belgacemi et al., 2020). SHH deprivation efficiency was confirmed by a 35% reduction in *GLI2* transcripts upon AB5E1 treatment (Supplementary Figure S1A). As we previously demonstrated, it induced a reduction of ciliogenesis, as evidenced by a 40% decrease in *FOXJ1* transcripts (Supplementary Figure S1B) and a reduction in multiciliated cells in the course of differentiation (Supplementary Figure S1C). The 1,596 genes that were significantly deregulated in AB5E1-treated cells (Figure 1A) are listed in Supplementary Material (Supplementary Table S2). Eighteen genes were deregulated more than 2-fold, five were downregulated (*CXCL5, DEFB4A, SFRP1, SLC5A8, and FCGBP*), and 13 were upregulated (*ASB14, C1QTNF8, CILP, COL11A2, FER1L5, HPX, LIMS2, MYH3, OVCH2, POU5F1, SULT1A2, TMEM52, and TNNI3*) (Figures 1A,B). The transcriptomic profile of these 18 genes at the single-cell level evidenced low proportions of lung cell populations expressing transcripts consistent with a biological role oriented towards differentiation. In immune and stromal cells, 1.10% ± 2.62% [0.001–10.18] and 2.50% ± 6.39% [0.01–26.87] of cells, respectively, expressed the 18 genes (Figure 1C). They were detected in 1.52% ± 2.72% [0.01–11.35] of pneumocytes and 1.89% ± 2% [0.12–5.69] of tracheobronchial epithelial cells. A gene ontology (GO) analysis revealed that all the statistically significant GO terms (biological process, cellular component, and annotated keywords) were associated with ciliogenesis and cilia structure in AECs (Figure 1D). This bioinformatics analysis prompted us to evaluate the master gene of differentiation, *POU5F1* (POU Class 5 Homeobox 1, also named OCT3/4), in COPD patients.

**FIGURE 2:**
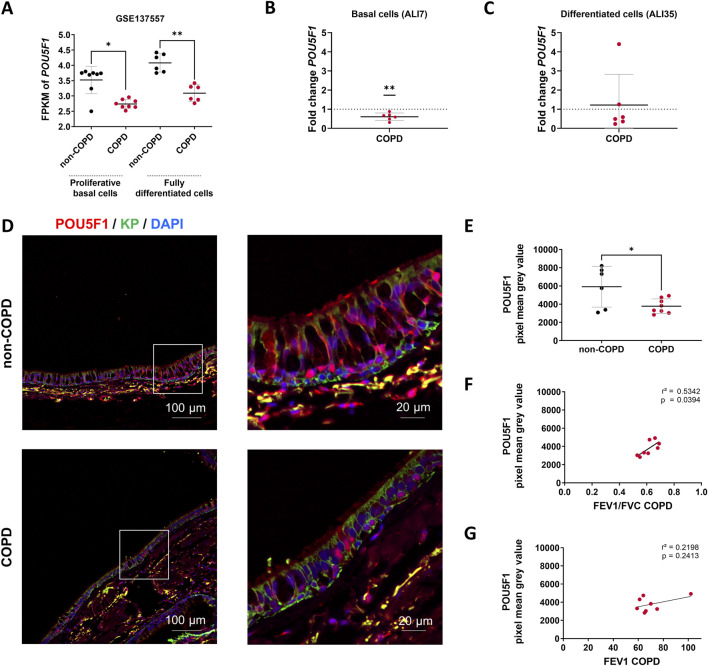
POU5F1 is altered in COPD patients. **(A)** Dot plot with mean ± SD showing *POU5F1* expression analysis from RNAseq data (GSE137557). The proliferative basal cells (n = 8) were collected for analysis before the air switch, and the fully differentiated cells (n = 6) were collected for analysis after 28 days of differentiation. *p < 0.05; **p < 0.01; non-COPD vs. COPD. FPKM: Fragments per kilobase million. **(B)** Dot plot with mean ± SD showing fold change according to the 2^−ΔΔCT^ method for the *POU5F1* expression analysis on basal cells and **(C)** differentiated cells. The average threshold cycle (Ct) values were respectively at ALI7 in non-COPD (n = 6) vs. COPD (n = 6) AECs: 22.5 Ct vs. 22 Ct for *GAPDH*, and 31.5 Ct vs. 31 Ct for *POU5F1*; at ALI35 in non-COPD vs. COPD AECs: 22.7 Ct vs. 23.2 Ct for *GAPDH*, and 31 Ct vs. 31.7 Ct for *POU5F1*. **p < 0.01; ns: non-significant; non-COPD vs. COPD. **(D)** Representative micrographs showing bronchial epithelia of non-COPD individuals and COPD patients immunostained for POU5F1 (red), epithelial cytokeratins (KP, green), and cell nuclei (DAPI, blue). Magnification corresponding to the selected area is shown. **(E)** Dot plot with mean ± SD representing POU5F1 pixel mean grey values of non-COPD (n = 6, black) individuals and COPD (n = 8, red) patients. *p < 0.05; non-COPD vs. COPD. **(F)** Linear regression representing a correlation between POU5F1 pixel mean gray levels and FEV_1_/FVC in bronchial epithelia of COPD patients (n = 8). **(G)** Linear regression representing a correlation between POU5F1 pixel mean gray levels and FEV_1_ in bronchial epithelia of COPD patients (n = 8).

First, we analyzed *POU5F1* transcripts in non-COPD individuals and COPD patients. In a public dataset (GSE137557) that includes patients with very severe COPD (FEV_1_% = 18.6 ± 5.1), there was a decrease of 22.3% in the transcript levels of *POU5F1* in proliferative basal cells (2.74 ± 0.147FPKM vs. 3.52 ± 0.45FPKM, COPD vs. non-COPD, p < 0.05) and a 24.2% reduction in fully differentiated cells (3.09 ± 0.27FPKM vs. 4.08 ± 0.3FPKM, COPD vs. non-COPD, p < 0.01) (Figure 2A). We analyzed *POU5F1* transcripts in AECs isolated from bronchial brushings of non-COPD individuals and patients with moderate COPD (FEV_1_% = 51.6 ± 21.5). There was a decrease of 40% in the transcript levels of *POU5F1* in COPD samples at ALI7 (Fold change = 0.6, COPD vs. non-COPD, p < 0.01) but no difference between non-COPD and COPD samples at ALI35 (fold change = 1.2) (Figures 2B,C). In addition, there was no correlation between FEV_1_ or FEV_1_/FVC in COPD patients and *POU5F1* transcript expression at ALI7 (*r* ^2^ = 0.0894, p > 0.05, and *r* ^2^ = 0.0032, p > 0.05, respectively) or at ALI35 (*r* ^2^ = 0.12, p > 0.05, and *r* ^2^ = 0.2303, p > 0.05, respectively) (Supplementary Figure S2). Finally, POU5F1 protein levels and localizations in the bronchial epithelia of FFPE lung tissues were investigated. Interestingly, POU5F1 protein epithelial localization was decreased by 36.1% in COPD patients (3783 ± 797.3 PMGV vs. 5912 ± 2233 PMGV COPD vs. non-COPD, p < 0.05) (Figures 2D,E). A low POU5F1 epithelial protein abundance was correlated with low FEV_1_/FVC in COPD patients (*r* ^2^ = 0.5342, p < 0.05) but not with the FEV_1_ (*r* ^2^ = 0.2198, p > 0.05) (Figures 2F,G). Altogether, these findings may suggest an association between POU5F1 levels and the severity of lung function impairment in COPD patients.

## 4 Discussion

Because epithelial remodeling may originate from alterations in early molecular and cellular events occurring during AEC differentiation, and we previously highlighted the crucial role of the Hedgehog pathway, we analyzed the transcriptomic print of non-differentiated AECs upon Hedgehog inhibition. This original experimental design seeking HH pathway regulators during AEC differentiation highlighted a list of potential key genes partially responsible for epithelial remodeling, especially regarding cilia homeostasis. Focusing on the reprogramming factor POU5F1, we demonstrated its alteration in COPD patients.

POU5F1 immunoreactivity in bronchial biopsy was already found to be significantly reduced and involved in lung epithelial cell plasticity in smoker/ex-smoker COPD patients compared to non-smoker, non-COPD individuals (Gagliardo et al., 2022). We confirmed these findings and established independence from smoking history because we matched the clinical features of our cohort.

An increase in *POU5F1* transcripts in the early steps of AEC differentiation, in the presence of AB5E1, suggests that HH pathway activation promotes the switch between proliferation and differentiation in respiratory basal cells, partly via *POU5F1*. This is supported by experimental findings in mouse embryonic stem cells (mESCs), in which reduced *POU5F1* levels stimulated mESC differentiation, while an overactivation of the HH pathway induced proliferation (Li et al., 2013). Similarly, *POU5F1* overexpression (DNA plasmid pCIG-Pou5f1) decreased HH pathway activation in mESC GLI1-activated cells (Li et al., 2016). Thereafter, the levels of *POU5F1* in COPD AECs are not sufficient to maintain a fully functional epithelium.

Despite the robust transcriptomic analysis of human AECs during differentiation and the validation of an intriguing association between POU5F1 and HH signaling in the respiratory context, our study presents some limitations. Although the deprivation of SHH via AB5E1 is efficient, residual activity of the HH pathway may remain. Even if the *in vitro* modulation of *POU5F1* is challenging because of its direct control of the epithelial stemness properties, AEC differentiation must be addressed in future experimental approaches to precisely identify the molecular mechanisms.

In conclusion, we demonstrated that interfering with HH signaling during AEC differentiation can help identify essential biological markers such as POU5F1, bridging this multifaceted pathway with epithelial plasticity and remodeling in the context of COPD. Additional investigations are required to fully characterize the complex molecular mechanisms at stake and explore other potential candidates.

## Data Availability

The datasets presented in this study can be found in online repositories. The names of the repository/repositories and accession number(s) can be found in the article/Supplementary Material.
