# Supplementary material for: POU5F1 bridges Hedgehog signaling and epithelial remodeling in COPD
Source: Front Cell Dev Biol. 2025 Jul 2;13:1566251. doi: 10.3389/fcell.2025.1566251 (PMC12263618; doi:10.3389/fcell.2025.1566251)
Supplement: Supplementary file 1 [file DataSheet1.docx]

**POU5F1 bridges Hedgehog signalling and epithelial remodelling in COPD**

**Supplementary material**

Supplementary figures:

Figure S1: Experimental validation of SHH inhibition with AB5E1 for the RNAseq analysis.

Figure S2: *POU5F1* transcript level is not correlated with clinical parameters in COPD patients

Supplementary tables:

Table S1: Clinical characteristics of patients from GSE137557.

Table S2: List of significantly deregulated genes in AB5E1-treated AEC.

**Supplementary figures**

**Figure S1: Experimental validation of SHH inhibition with AB5E1 for the RNAseq analysis.**

Dot plots with mean ±SD showing fold changes upon AB5E1 treatment according to the 2^-ΔΔCT^ method for *GLI2* (**A**) and *FOXJ1* (**B**) transcript level analysis on basal cells. ALI cultures were performed from isolated AEC obtained from non-COPD individuals (**Table 2**, n=5). *p<0.05; AB5E1-treated AEC vs non-treated AEC at ALI7. (**C**) Representative micrographs showing AEC treated with AB5E1 or not at ALI14 immunostained for ARL13B (red, multiciliated cells), and cell nuclei (DAPI, blue).


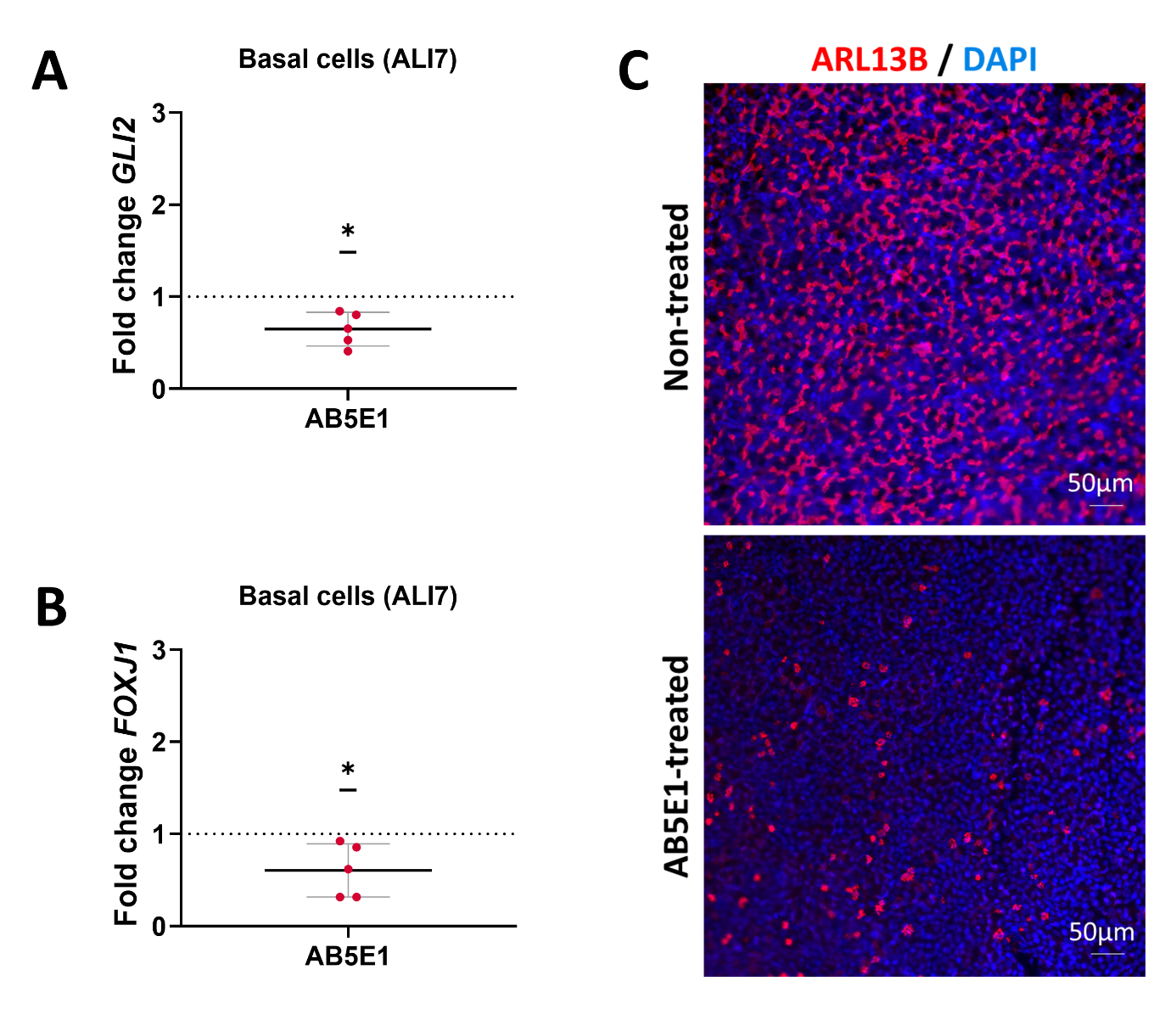


**Figure S2: *POU5F1*** **transcript level is not correlated with clinical parameters in COPD patients**

Linear regressions representing the correlations between the fold changes of *POU5F1* transcript levels and FEV_1_/FVC or FEV_1_ in ALI7 (A, B) and in ALI35 (C, D) AEC cultures of COPD patients.


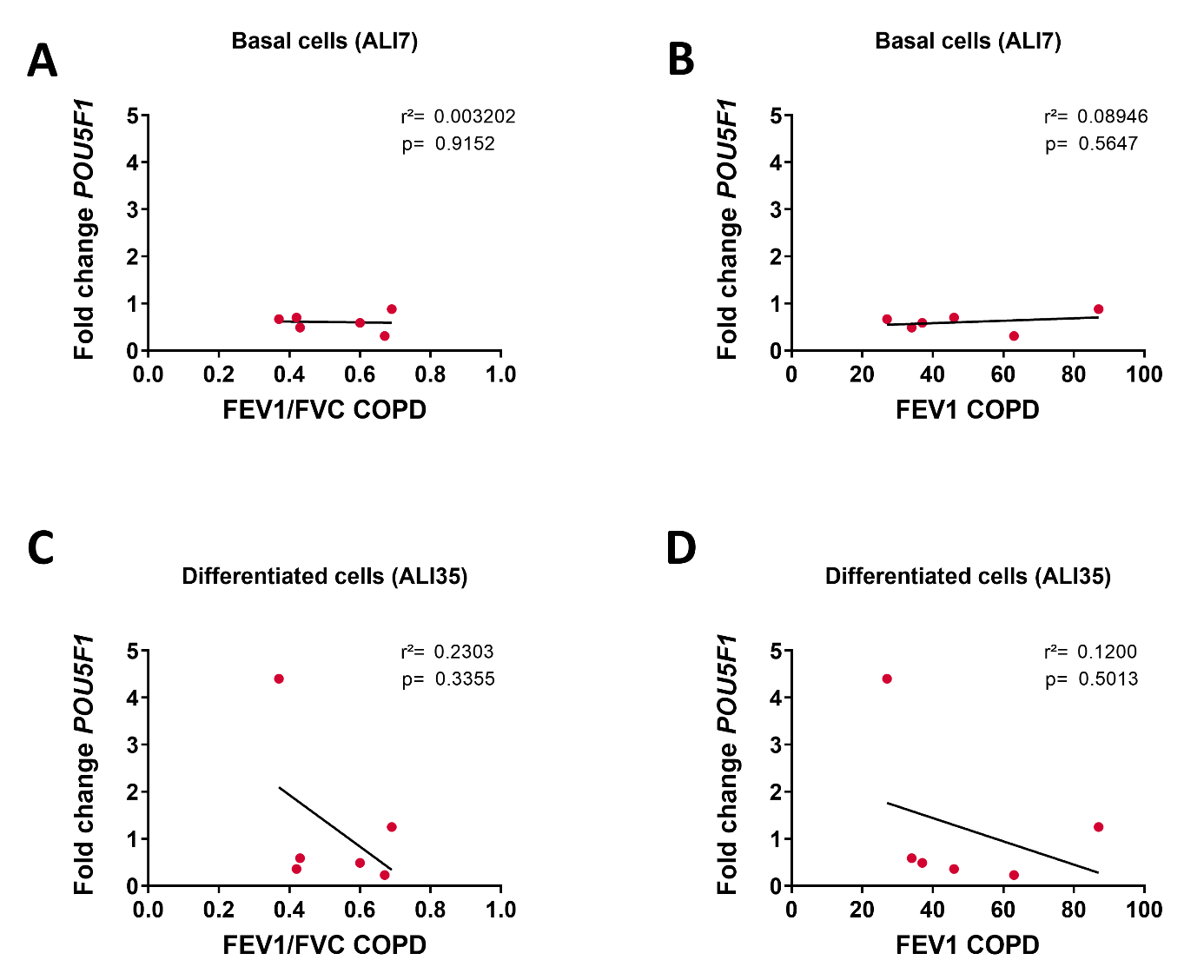


**Supplementary tables**

**Table S1: Clinical characteristics of patients from GSE137557.**

Data are expressed as mean±SD [min-max], number, or percentage. FEV_1_: Forced Expiratory Volume in 1s; FVC: Forced Vital Capacity.

|  | **GSE137557** | |
| --- | --- | --- |
|  | **Non-COPD** | **COPD** |
| Number of subjects | 8 | 8 |
| Gender F/M | 4/4 | 3/5 |
| Age, years | 46.3±15 [16-62] | 55.1±7.7 [45-67] |
|  |  |  |
| **Smoking history** |  |  |
| Never | 8 | 0 |
| Former | 0 | 8 |
| Current | 0 | 0 |
| Pack-years |  | 57.5±39.2 [25-136] |
|  |  |  |
| **Lung functional parameters** |  |  |
| FEV_1_ % pred | NA | 18.6±5.1 [13-29] |
| FEV_1_/FVC | NA | NA |

**Table S2: List of significantly deregulated genes in AB5E1-treated AEC.**

| *GENES* | BaseMean | Log2FoldChange  (AB5E1/CTL) | p-value | Adjusted p-value |
| --- | --- | --- | --- | --- |
| *CXCL5* | 2000.546 | -1.472 | 0.0015 | 0.0220 |
| *DEFB4A* | 73.089 | -1.252 | 0.0007 | 0.0132 |
| *SFRP1* | 44.402 | -1.148 | 0.0020 | 0.0270 |
| *SLC5A8* | 404.704 | -1.094 | 0.0007 | 0.0139 |
| *FCGBP* | 2756.670 | -1.031 | 0.0011 | 0.0183 |
| *CLEC7A* | 49.198 | -0.891 | 0.0054 | 0.0483 |
| *ABCA12* | 519.498 | -0.891 | 5.68E-09 | 4.18E-06 |
| *CDH6* | 731.345 | -0.875 | 0.0011 | 0.0176 |
| *CEACAM5* | 1314.608 | -0.873 | 7.00E-15 | 3.27E-11 |
| *TMEM178B* | 176.066 | -0.847 | 1.38E-05 | 0.0012 |
| *ALDH1L2* | 83.012 | -0.831 | 0.0005 | 0.0112 |
| *MUC21* | 80.305 | -0.830 | 0.0020 | 0.0268 |
| *PAX8* | 68.900 | -0.824 | 0.0014 | 0.0217 |
| *ARG2* | 56.526 | -0.795 | 0.0041 | 0.0410 |
| *RGS7BP* | 87.079 | -0.793 | 0.0007 | 0.0137 |
| *POMK* | 54.232 | -0.786 | 0.0036 | 0.0379 |
| *KIAA1211* | 64.461 | -0.764 | 0.0037 | 0.0389 |
| *NKX3-1* | 370.072 | -0.739 | 9.54E-05 | 0.0039 |
| *MTNR1A* | 119.880 | -0.726 | 0.0013 | 0.0197 |
| *ETV5* | 84.233 | -0.724 | 0.0036 | 0.0384 |
| *GPNMB* | 732.942 | -0.699 | 3.86E-06 | 0.0005 |
| *C8orf4* | 645.671 | -0.694 | 3.81E-06 | 0.0005 |
| *ERCC6L2* | 144.766 | -0.691 | 0.0001 | 0.0047 |
| *KIAA0754* | 299.748 | -0.690 | 1.33E-06 | 0.0002 |
| *DDI2* | 184.748 | -0.683 | 1.17E-05 | 0.0010 |
| *CHL1* | 805.060 | -0.679 | 7.95E-06 | 0.0008 |
| *RASGRP1* | 83.799 | -0.673 | 0.0035 | 0.0376 |
| *MYEOV* | 129.610 | -0.644 | 0.0019 | 0.0261 |
| *PCDHAC2* | 226.713 | -0.636 | 0.0002 | 0.0061 |
| *KMO* | 154.226 | -0.636 | 0.0047 | 0.0444 |
| *DUSP5* | 624.312 | -0.630 | 2.08E-06 | 0.0003 |
| *EFCAB4B* | 550.389 | -0.626 | 3.31E-05 | 0.0020 |
| *CSNK2A3* | 158.225 | -0.605 | 0.0012 | 0.0184 |
| *LBH* | 406.220 | -0.591 | 2.92E-05 | 0.0018 |
| *BAALC* | 176.452 | -0.586 | 0.0010 | 0.0168 |
| *MPV17L* | 199.960 | -0.583 | 0.0014 | 0.0210 |
| *NLGN4X* | 231.636 | -0.581 | 0.0008 | 0.0145 |
| *VCAN* | 903.977 | -0.566 | 2.59E-06 | 0.0004 |
| *ANK1* | 130.067 | -0.565 | 0.0040 | 0.0403 |
| *C2* | 319.438 | -0.558 | 2.50E-05 | 0.0017 |
| *ATP12A* | 3847.442 | -0.538 | 1.27E-06 | 0.0002 |
| *GLIPR1* | 198.472 | -0.538 | 0.0057 | 0.0499 |
| *FAM198B* | 210.598 | -0.538 | 0.0002 | 0.0064 |
| *RC3H2* | 958.348 | -0.535 | 7.52E-06 | 0.0008 |
| *IRAK2* | 547.392 | -0.532 | 1.85E-05 | 0.0014 |
| *HCAR1* | 260.575 | -0.528 | 0.0003 | 0.0076 |
| *IDO1* | 1077.110 | -0.527 | 5.96E-05 | 0.0028 |
| *TRNR* | 207.433 | -0.523 | 0.0019 | 0.0259 |
| *SNX29* | 332.482 | -0.520 | 0.0009 | 0.0161 |
| *ZSWIM5* | 135.587 | -0.519 | 0.0030 | 0.0346 |
| *PTPLB* | 396.744 | -0.515 | 0.0004 | 0.0099 |
| *FAM131B* | 296.313 | -0.511 | 0.0003 | 0.0083 |
| *LOC101927371* | 143.559 | -0.510 | 0.0039 | 0.0396 |
| *FRK* | 408.444 | -0.510 | 0.0002 | 0.0058 |
| *CD36* | 275.950 | -0.507 | 0.0002 | 0.0073 |
| *LNPEP* | 529.421 | -0.501 | 6.01E-06 | 0.0007 |
| *IPO11* | 407.723 | -0.500 | 0.0004 | 0.0105 |
| *CDH13* | 301.991 | -0.499 | 0.0013 | 0.0200 |
| *MYL9* | 245.889 | -0.496 | 0.0035 | 0.0376 |
| *HS6ST2* | 409.028 | -0.493 | 3.23E-05 | 0.0019 |
| *MARVELD2* | 1004.580 | -0.490 | 2.54E-06 | 0.0004 |
| *MMP2* | 1452.286 | -0.488 | 7.68E-05 | 0.0033 |
| *PCDHGC3* | 539.807 | -0.483 | 0.0001 | 0.0052 |
| *PRRX2* | 297.115 | -0.478 | 0.0034 | 0.0371 |
| *MAP2* | 329.339 | -0.469 | 0.0030 | 0.0344 |
| *FAM169A* | 418.632 | -0.469 | 0.0002 | 0.0067 |
| *SOD2* | 3840.817 | -0.468 | 6.57E-05 | 0.0030 |
| *RCAN1* | 1523.258 | -0.464 | 7.19E-05 | 0.0032 |
| *RSC1A1* | 191.990 | -0.463 | 0.0033 | 0.0361 |
| *GCNT3* | 1409.792 | -0.462 | 8.75E-05 | 0.0037 |
| *IGF2R* | 5227.108 | -0.456 | 1.12E-08 | 6.79E-06 |
| *DKK3* | 1308.648 | -0.452 | 0.0005 | 0.0116 |
| *SLITRK6* | 3317.063 | -0.446 | 7.28E-08 | 2.75E-05 |
| *PDZD8* | 1130.049 | -0.443 | 1.92E-05 | 0.0014 |
| *PANK3* | 1072.663 | -0.443 | 6.32E-06 | 0.0007 |
| *RGP1* | 844.681 | -0.440 | 7.26E-05 | 0.0032 |
| *ALPL* | 2206.735 | -0.439 | 0.0005 | 0.0106 |
| *LRP12* | 592.613 | -0.436 | 0.0006 | 0.0123 |
| *ITGB6* | 3772.086 | -0.435 | 8.20E-08 | 3.02E-05 |
| *SLC30A1* | 530.568 | -0.434 | 0.0001 | 0.0047 |
| *MMGT1* | 1103.199 | -0.429 | 0.0002 | 0.0063 |
| *EGFR* | 2973.418 | -0.429 | 2.76E-08 | 1.43E-05 |
| *ANAPC1* | 687.228 | -0.425 | 9.76E-06 | 0.0009 |
| *SOWAHB* | 670.109 | -0.421 | 0.0001 | 0.0043 |
| *PCDH20* | 2135.773 | -0.421 | 8.45E-06 | 0.0008 |
| *ABCC4* | 495.455 | -0.414 | 0.0009 | 0.0155 |
| *UTP20* | 1281.630 | -0.412 | 7.41E-05 | 0.0033 |
| *TMEM164* | 679.587 | -0.409 | 0.0002 | 0.0069 |
| *HDAC9* | 1089.284 | -0.408 | 0.0041 | 0.0409 |
| *SLFN5* | 4029.749 | -0.408 | 0.0001 | 0.0048 |
| *THBS1* | 926.422 | -0.407 | 0.0012 | 0.0190 |
| *SLC7A2* | 596.139 | -0.405 | 0.0022 | 0.0288 |
| *UBASH3B* | 362.109 | -0.400 | 0.0008 | 0.0146 |
| *CPD* | 25935.800 | -0.397 | 3.40E-06 | 0.0005 |
| *SLC12A2* | 3636.172 | -0.396 | 3.47E-05 | 0.0020 |
| *ARHGAP42* | 439.305 | -0.395 | 0.0009 | 0.0155 |
| *FAM83G* | 484.752 | -0.393 | 0.0009 | 0.0156 |
| *CLIC4* | 5295.251 | -0.391 | 1.21E-06 | 0.0002 |
| *ARHGAP26* | 1283.902 | -0.386 | 0.0001 | 0.0041 |
| *ABHD2* | 7785.214 | -0.385 | 3.89E-07 | 9.96E-05 |
| *UHRF1BP1L* | 613.598 | -0.383 | 0.0008 | 0.0146 |
| *MGAT4A* | 847.475 | -0.383 | 4.44E-05 | 0.0023 |
| *SRD5A3* | 2502.035 | -0.382 | 0.0004 | 0.0100 |
| *ITGA2* | 6531.945 | -0.381 | 9.04E-08 | 3.24E-05 |
| *GTF3C4* | 680.178 | -0.381 | 0.0009 | 0.0156 |
| *TMCO3* | 3381.281 | -0.381 | 9.91E-05 | 0.0041 |
| *TBC1D9* | 1763.477 | -0.380 | 2.16E-06 | 0.0003 |
| *HPGD* | 704.542 | -0.380 | 0.0010 | 0.0172 |
| *REL* | 322.556 | -0.379 | 0.0055 | 0.0491 |
| *NUP155* | 723.898 | -0.379 | 0.0002 | 0.0071 |
| *NOMO2* | 615.200 | -0.379 | 0.0014 | 0.0215 |
| *CLCA2* | 12399.209 | -0.378 | 6.79E-05 | 0.0031 |
| *TNC* | 6573.295 | -0.378 | 2.38E-05 | 0.0016 |
| *SLC29A1* | 245.242 | -0.378 | 0.0053 | 0.0482 |
| *FAM83F* | 760.077 | -0.377 | 0.0005 | 0.0112 |
| *CHST4* | 877.815 | -0.376 | 0.0020 | 0.0269 |
| *SEMA5A* | 1906.753 | -0.376 | 6.06E-06 | 0.0007 |
| *TFRC* | 2332.286 | -0.375 | 4.01E-05 | 0.0022 |
| *SCRN1* | 4062.674 | -0.374 | 4.45E-06 | 0.0006 |
| *PMEPA1* | 1447.469 | -0.373 | 0.0001 | 0.0048 |
| *ID4* | 468.743 | -0.372 | 0.0028 | 0.0333 |
| *HEG1* | 327.984 | -0.371 | 0.0039 | 0.0396 |
| *EDIL3* | 413.473 | -0.371 | 0.0048 | 0.0449 |
| *KIF2A* | 1776.567 | -0.369 | 0.0004 | 0.0094 |
| *PLEKHG1* | 969.089 | -0.369 | 0.0005 | 0.0106 |
| *CD109* | 4334.330 | -0.368 | 4.23E-05 | 0.0023 |
| *CLCN4* | 322.947 | -0.365 | 0.0037 | 0.0388 |
| *NRP2* | 1883.452 | -0.363 | 0.0003 | 0.0088 |
| *SERINC3* | 3609.293 | -0.362 | 1.95E-05 | 0.0014 |
| *ZC3H12C* | 1158.976 | -0.362 | 0.0002 | 0.0057 |
| *TMEM184C* | 1266.995 | -0.361 | 0.0004 | 0.0104 |
| *GALNT14* | 564.726 | -0.361 | 0.0008 | 0.0146 |
| *NTN1* | 6859.113 | -0.360 | 1.50E-07 | 4.88E-05 |
| *NUAK1* | 685.053 | -0.360 | 0.0002 | 0.0069 |
| *SDK2* | 851.828 | -0.358 | 0.0004 | 0.0101 |
| *GCC1* | 991.490 | -0.357 | 0.0001 | 0.0051 |
| *B4GALT5* | 12529.394 | -0.357 | 9.23E-07 | 0.0002 |
| *DOC2B* | 1350.321 | -0.356 | 0.0014 | 0.0215 |
| *FERMT1* | 1415.878 | -0.356 | 4.81E-05 | 0.0025 |
| *SLC6A14* | 12269.646 | -0.355 | 3.84E-05 | 0.0022 |
| *OPHN1* | 378.402 | -0.355 | 0.0019 | 0.0266 |
| *KCNE3* | 1701.619 | -0.355 | 2.80E-05 | 0.0018 |
| *ZBTB38* | 2463.705 | -0.354 | 1.36E-05 | 0.0012 |
| *GPRC5B* | 1051.716 | -0.351 | 0.0001 | 0.0051 |
| *NBEA* | 1033.722 | -0.350 | 0.0015 | 0.0222 |
| *LRIG3* | 2509.802 | -0.350 | 6.15E-07 | 0.0001 |
| *RAB32* | 312.008 | -0.349 | 0.0039 | 0.0398 |
| *PTPRK* | 4495.953 | -0.349 | 3.18E-05 | 0.0019 |
| *TGFB2* | 2680.664 | -0.349 | 0.0001 | 0.0052 |
| *SLC39A6* | 3579.981 | -0.346 | 0.0001 | 0.0049 |
| *C12orf49* | 1753.027 | -0.346 | 0.0002 | 0.0071 |
| *ITGAV* | 5772.029 | -0.345 | 2.06E-05 | 0.0015 |
| *ADAM9* | 12270.681 | -0.345 | 2.43E-05 | 0.0016 |
| *LMBR1* | 1548.952 | -0.345 | 0.0003 | 0.0079 |
| *LIPH* | 729.862 | -0.344 | 0.0006 | 0.0117 |
| *LPIN2* | 513.135 | -0.342 | 0.0033 | 0.0361 |
| *MGAT5* | 2275.623 | -0.342 | 9.10E-05 | 0.0038 |
| *LAYN* | 815.387 | -0.342 | 0.0006 | 0.0117 |
| *SERINC5* | 3164.268 | -0.339 | 4.83E-06 | 0.0006 |
| *IRS1* | 1986.254 | -0.339 | 0.0008 | 0.0154 |
| *PCDH1* | 4637.236 | -0.339 | 0.0001 | 0.0051 |
| *CLIP2* | 387.574 | -0.338 | 0.0030 | 0.0346 |
| *GNPTAB* | 2472.745 | -0.338 | 2.46E-05 | 0.0016 |
| *CRB3* | 608.476 | -0.338 | 0.0027 | 0.0324 |
| *DOPEY2* | 952.720 | -0.338 | 0.0001 | 0.0047 |
| *KBTBD7* | 320.808 | -0.337 | 0.0052 | 0.0476 |
| *ACVR1* | 1468.267 | -0.337 | 3.34E-05 | 0.0020 |
| *MAML3* | 603.238 | -0.337 | 0.0009 | 0.0157 |
| *RNF24* | 877.529 | -0.336 | 0.0004 | 0.0094 |
| *CSNK1A1* | 9248.856 | -0.335 | 5.03E-05 | 0.0025 |
| *CEACAM6* | 23736.439 | -0.335 | 0.0003 | 0.0085 |
| *FAM84A* | 3078.549 | -0.335 | 1.63E-05 | 0.0013 |
| *ITGB1* | 16041.696 | -0.333 | 0.0005 | 0.0113 |
| *RHOB* | 1447.007 | -0.332 | 9.52E-05 | 0.0039 |
| *GTF2I* | 3099.153 | -0.332 | 0.0002 | 0.0068 |
| *SNX18* | 1872.749 | -0.331 | 3.27E-05 | 0.0019 |
| *G3BP2* | 4945.365 | -0.331 | 3.88E-05 | 0.0022 |
| *SLC40A1* | 1068.386 | -0.331 | 0.0019 | 0.0262 |
| *ENDOD1* | 1669.684 | -0.331 | 0.0002 | 0.0063 |
| *EMP1* | 1721.574 | -0.331 | 0.0036 | 0.0380 |
| *PXDN* | 4403.720 | -0.330 | 5.12E-06 | 0.0006 |
| *BMP3* | 1008.384 | -0.329 | 0.0009 | 0.0160 |
| *VEZF1* | 2692.726 | -0.329 | 1.86E-05 | 0.0014 |
| *MOB1A* | 1811.427 | -0.329 | 0.0005 | 0.0112 |
| *SLC16A7* | 1360.415 | -0.329 | 0.0027 | 0.0321 |
| *CYP51A1* | 4268.157 | -0.328 | 0.0028 | 0.0333 |
| *POF1B* | 1397.978 | -0.327 | 0.0001 | 0.0046 |
| *IGSF3* | 2444.111 | -0.327 | 2.42E-06 | 0.0004 |
| *SYT7* | 775.011 | -0.327 | 0.0004 | 0.0101 |
| *ACBD3* | 2490.568 | -0.327 | 6.50E-05 | 0.0030 |
| *KLF4* | 1886.606 | -0.326 | 3.03E-05 | 0.0019 |
| *KRT23* | 3661.703 | -0.325 | 5.47E-05 | 0.0027 |
| *ASCC3* | 3027.420 | -0.324 | 6.70E-05 | 0.0031 |
| *PPAP2B* | 873.703 | -0.323 | 0.0024 | 0.0300 |
| *SLC7A1* | 2510.184 | -0.323 | 0.0004 | 0.0103 |
| *DOCK7* | 1629.332 | -0.322 | 0.0001 | 0.0047 |
| *ITGB5* | 1842.760 | -0.321 | 0.0005 | 0.0106 |
| *SEC23B* | 2625.011 | -0.321 | 0.0005 | 0.0107 |
| *CDYL* | 1046.347 | -0.320 | 0.0005 | 0.0106 |
| *EPAS1* | 28776.026 | -0.320 | 2.34E-07 | 6.42E-05 |
| *ZDHHC2* | 1426.061 | -0.320 | 0.0001 | 0.0048 |
| *SH3PXD2B* | 3446.315 | -0.319 | 4.10E-05 | 0.0022 |
| *PJA2* | 3201.758 | -0.318 | 0.0001 | 0.0047 |
| *SNN* | 1466.265 | -0.318 | 0.0002 | 0.0061 |
| *MTMR6* | 1442.643 | -0.317 | 0.0006 | 0.0117 |
| *BDKRB2* | 483.497 | -0.317 | 0.0037 | 0.0388 |
| *PPM1L* | 434.953 | -0.317 | 0.0047 | 0.0444 |
| *RNF145* | 10416.190 | -0.317 | 9.39E-05 | 0.0039 |
| *DSE* | 1725.280 | -0.317 | 0.0008 | 0.0153 |
| *MTMR12* | 1709.609 | -0.316 | 5.94E-05 | 0.0028 |
| *ZFP36L2* | 2401.154 | -0.316 | 0.0002 | 0.0073 |
| *LITAF* | 8140.152 | -0.316 | 0.0003 | 0.0086 |
| *PLBD2* | 726.579 | -0.315 | 0.0006 | 0.0126 |
| *CCND1* | 12496.278 | -0.315 | 6.44E-06 | 0.0007 |
| *ZFYVE9* | 593.175 | -0.315 | 0.0054 | 0.0483 |
| *RPS6KA5* | 436.981 | -0.315 | 0.0055 | 0.0491 |
| *RFC1* | 1771.952 | -0.314 | 0.0002 | 0.0060 |
| *MFHAS1* | 683.166 | -0.314 | 0.0022 | 0.0284 |
| *ATP11C* | 711.148 | -0.314 | 0.0022 | 0.0288 |
| *MID2* | 932.045 | -0.313 | 0.0013 | 0.0202 |
| *LRBA* | 4033.265 | -0.313 | 0.0001 | 0.0047 |
| *WDR7* | 828.040 | -0.313 | 0.0023 | 0.0289 |
| *GALNT5* | 1545.490 | -0.312 | 0.0003 | 0.0081 |
| *PIK3CA* | 683.883 | -0.311 | 0.0031 | 0.0353 |
| *MGLL* | 787.278 | -0.311 | 0.0044 | 0.0424 |
| *ZXDB* | 477.409 | -0.311 | 0.0033 | 0.0360 |
| *OSBPL8* | 2159.982 | -0.311 | 0.0003 | 0.0083 |
| *CADPS2* | 1329.840 | -0.311 | 0.0012 | 0.0190 |
| *MANSC1* | 2925.774 | -0.311 | 0.0002 | 0.0061 |
| *LIPA* | 3177.611 | -0.311 | 0.0005 | 0.0106 |
| *KIAA1671* | 5622.266 | -0.311 | 1.01E-05 | 0.0009 |
| *SDK1* | 1844.161 | -0.310 | 5.43E-05 | 0.0027 |
| *ZNF703* | 1730.821 | -0.310 | 0.0011 | 0.0176 |
| *WASL* | 3664.850 | -0.309 | 8.34E-05 | 0.0036 |
| *GNG12* | 4741.618 | -0.307 | 0.0003 | 0.0074 |
| *SLC35D1* | 1078.140 | -0.306 | 0.0002 | 0.0064 |
| *MEIS3P1* | 586.292 | -0.306 | 0.0023 | 0.0291 |
| *CAMK1D* | 2294.809 | -0.305 | 5.96E-05 | 0.0028 |
| *MBTPS2* | 779.897 | -0.305 | 0.0026 | 0.0317 |
| *C16orf72* | 1658.104 | -0.305 | 0.0005 | 0.0112 |
| *TBC1D5* | 1759.775 | -0.304 | 6.39E-05 | 0.0030 |
| *PRKCH* | 931.764 | -0.304 | 0.0013 | 0.0201 |
| *FNDC3B* | 3671.524 | -0.304 | 3.72E-05 | 0.0021 |
| *RAB27B* | 2855.694 | -0.304 | 6.72E-05 | 0.0031 |
| *SOCS5* | 688.077 | -0.303 | 0.0028 | 0.0334 |
| *EIF2AK3* | 1527.974 | -0.303 | 0.0002 | 0.0061 |
| *HECA* | 1133.239 | -0.303 | 0.0005 | 0.0116 |
| *CNOT1* | 7939.220 | -0.302 | 4.02E-05 | 0.0022 |
| *MCFD2* | 2431.281 | -0.302 | 0.0002 | 0.0061 |
| *FYCO1* | 2500.312 | -0.302 | 0.0002 | 0.0058 |
| *GFM1* | 1711.304 | -0.302 | 0.0008 | 0.0148 |
| *TRIM14* | 2064.795 | -0.301 | 0.0005 | 0.0117 |
| *LTBP1* | 692.446 | -0.300 | 0.0030 | 0.0345 |
| *MPP7* | 1962.193 | -0.299 | 0.0004 | 0.0101 |
| *SEMA3C* | 6767.126 | -0.299 | 0.0002 | 0.0061 |
| *ATP9A* | 4463.870 | -0.299 | 1.41E-05 | 0.0012 |
| *GNA13* | 2155.037 | -0.299 | 4.06E-05 | 0.0022 |
| *TRNL1* | 3208.105 | -0.299 | 0.0009 | 0.0158 |
| *PPTC7* | 1798.596 | -0.299 | 0.0007 | 0.0132 |
| *PTPN9* | 1316.895 | -0.299 | 0.0009 | 0.0162 |
| *ZHX2* | 624.709 | -0.298 | 0.0029 | 0.0340 |
| *VAT1* | 2036.699 | -0.298 | 0.0002 | 0.0068 |
| *CHTF8* | 3781.271 | -0.298 | 0.0004 | 0.0099 |
| *SEC23A* | 1282.807 | -0.297 | 0.0009 | 0.0155 |
| *GPR107* | 4149.020 | -0.297 | 0.0001 | 0.0053 |
| *USP38* | 1195.726 | -0.296 | 0.0008 | 0.0153 |
| *PLEKHB2* | 5071.552 | -0.296 | 0.0027 | 0.0324 |
| *TRAPPC10* | 1103.710 | -0.295 | 0.0016 | 0.0237 |
| *CCSER2* | 1550.586 | -0.295 | 0.0003 | 0.0080 |
| *DAG1* | 5152.456 | -0.295 | 4.58E-05 | 0.0024 |
| *TEAD1* | 2278.412 | -0.294 | 0.0004 | 0.0093 |
| *NEDD9* | 450.708 | -0.293 | 0.0054 | 0.0485 |
| *SLC31A1* | 5079.126 | -0.293 | 0.0016 | 0.0232 |
| *CLDN1* | 8307.988 | -0.293 | 4.24E-06 | 0.0006 |
| *EIF4B* | 15826.901 | -0.293 | 4.39E-06 | 0.0006 |
| *PIGR* | 83137.644 | -0.293 | 0.0002 | 0.0067 |
| *SPTLC2* | 3400.599 | -0.292 | 0.0003 | 0.0080 |
| *MED20* | 643.679 | -0.292 | 0.0047 | 0.0444 |
| *KIAA1715* | 891.358 | -0.292 | 0.0011 | 0.0178 |
| *HEY1* | 1772.628 | -0.292 | 0.0007 | 0.0142 |
| *STK38L* | 1805.514 | -0.292 | 0.0006 | 0.0124 |
| *DSC3* | 4245.326 | -0.291 | 0.0004 | 0.0095 |
| *SLC20A2* | 2127.427 | -0.291 | 0.0007 | 0.0136 |
| *REV3L* | 1383.772 | -0.291 | 0.0009 | 0.0155 |
| *SETD7* | 2916.739 | -0.291 | 0.0006 | 0.0117 |
| *NEO1* | 3309.391 | -0.290 | 7.53E-05 | 0.0033 |
| *AHCYL2* | 2233.845 | -0.290 | 0.0024 | 0.0299 |
| *KIAA1324* | 2969.010 | -0.290 | 0.0038 | 0.0391 |
| *PPP4R2* | 1183.450 | -0.290 | 0.0006 | 0.0131 |
| *MAPK6* | 2960.507 | -0.289 | 0.0009 | 0.0155 |
| *SEPT11* | 3165.507 | -0.289 | 0.0003 | 0.0086 |
| *CLCN3* | 3324.643 | -0.289 | 0.0003 | 0.0080 |
| *TMOD3* | 4301.580 | -0.289 | 0.0009 | 0.0159 |
| *INPP5D* | 1490.709 | -0.289 | 0.0038 | 0.0392 |
| *FAT1* | 7780.212 | -0.289 | 5.30E-05 | 0.0026 |
| *IRS2* | 1217.776 | -0.289 | 0.0006 | 0.0127 |
| *ALDH4A1* | 1557.079 | -0.289 | 0.0005 | 0.0108 |
| *SMCR8* | 1373.239 | -0.288 | 0.0008 | 0.0146 |
| *BMPR2* | 2943.122 | -0.288 | 5.50E-05 | 0.0027 |
| *PIK3CB* | 1451.914 | -0.288 | 0.0013 | 0.0201 |
| *KRT80* | 881.620 | -0.288 | 0.0044 | 0.0429 |
| *HMGCR* | 4387.655 | -0.288 | 0.0043 | 0.0422 |
| *UBQLN1* | 4165.959 | -0.288 | 0.0002 | 0.0068 |
| *SEL1L* | 2992.011 | -0.287 | 0.0003 | 0.0086 |
| *EPHB4* | 4788.886 | -0.287 | 1.25E-05 | 0.0011 |
| *IDE* | 2546.221 | -0.287 | 0.0008 | 0.0153 |
| *FNIP1* | 778.376 | -0.286 | 0.0042 | 0.0416 |
| *ANO6* | 2054.330 | -0.286 | 0.0005 | 0.0113 |
| *MTF1* | 1521.944 | -0.286 | 0.0007 | 0.0143 |
| *KIF16B* | 942.979 | -0.285 | 0.0021 | 0.0277 |
| *GARS* | 3038.536 | -0.285 | 0.0040 | 0.0403 |
| *TGFBR1* | 1610.585 | -0.285 | 0.0003 | 0.0083 |
| *OSBPL11* | 746.711 | -0.284 | 0.0036 | 0.0380 |
| *RALGAPA2* | 5680.433 | -0.284 | 0.0003 | 0.0080 |
| *DENND4A* | 908.149 | -0.284 | 0.0038 | 0.0392 |
| *EXTL3* | 2194.796 | -0.284 | 0.0004 | 0.0098 |
| *LRP10* | 11082.561 | -0.283 | 0.0008 | 0.0145 |
| *RRAGC* | 803.604 | -0.283 | 0.0030 | 0.0344 |
| *MIPEP* | 1045.169 | -0.283 | 0.0018 | 0.0252 |
| *PLXDC2* | 833.915 | -0.283 | 0.0032 | 0.0355 |
| *DYNLL2* | 2601.708 | -0.282 | 0.0011 | 0.0183 |
| *SLC2A9* | 649.176 | -0.281 | 0.0028 | 0.0330 |
| *MFAP3* | 1501.165 | -0.281 | 0.0010 | 0.0164 |
| *MBP* | 3248.294 | -0.281 | 0.0003 | 0.0088 |
| *ADNP2* | 1194.663 | -0.280 | 0.0016 | 0.0228 |
| *OIP5-AS1* | 1037.743 | -0.280 | 0.0030 | 0.0342 |
| *ZFP64* | 733.578 | -0.280 | 0.0021 | 0.0276 |
| *MAPK1* | 3860.205 | -0.280 | 0.0004 | 0.0104 |
| *NR1D2* | 1415.737 | -0.280 | 0.0004 | 0.0105 |
| *COBLL1* | 1250.228 | -0.279 | 0.0014 | 0.0217 |
| *CERS6* | 4923.420 | -0.279 | 0.0001 | 0.0051 |
| *LMAN1* | 6256.915 | -0.279 | 0.0004 | 0.0095 |
| *SIK2* | 913.374 | -0.278 | 0.0031 | 0.0353 |
| *RNF141* | 2491.778 | -0.278 | 0.0005 | 0.0106 |
| *LUZP1* | 2033.081 | -0.277 | 0.0003 | 0.0080 |
| *ARHGAP35* | 3955.649 | -0.277 | 0.0003 | 0.0080 |
| *RHOQ* | 2349.891 | -0.277 | 0.0004 | 0.0105 |
| *FBXW11* | 2169.674 | -0.276 | 0.0009 | 0.0160 |
| *ATP10B* | 1516.217 | -0.276 | 0.0013 | 0.0197 |
| *SLC25A25* | 736.882 | -0.276 | 0.0025 | 0.0308 |
| *GNB1* | 8960.229 | -0.276 | 0.0003 | 0.0082 |
| *EPRS* | 4240.074 | -0.276 | 0.0008 | 0.0146 |
| *TMEM106B* | 3026.340 | -0.276 | 0.0003 | 0.0073 |
| *KLF13* | 3478.065 | -0.276 | 0.0011 | 0.0183 |
| *FEM1C* | 1313.299 | -0.275 | 0.0018 | 0.0258 |
| *PARG* | 1052.221 | -0.275 | 0.0051 | 0.0469 |
| *GNAI1* | 3349.781 | -0.275 | 5.74E-05 | 0.0028 |
| *ARFGEF1* | 4123.054 | -0.275 | 0.0005 | 0.0106 |
| *ATP7A* | 832.086 | -0.274 | 0.0021 | 0.0277 |
| *EXOC4* | 2540.144 | -0.274 | 0.0031 | 0.0353 |
| *VPS26A* | 2686.503 | -0.274 | 0.0008 | 0.0154 |
| *PIK3R3* | 5557.420 | -0.274 | 5.65E-05 | 0.0027 |
| *RAPGEF1* | 2199.902 | -0.274 | 0.0003 | 0.0079 |
| *UBTD2* | 1349.425 | -0.274 | 0.0019 | 0.0261 |
| *CALU* | 3615.580 | -0.274 | 0.0006 | 0.0127 |
| *SEPN1* | 5366.695 | -0.274 | 8.74E-05 | 0.0037 |
| *GMFB* | 1395.112 | -0.274 | 0.0009 | 0.0158 |
| *TAB2* | 2800.981 | -0.274 | 0.0008 | 0.0152 |
| *SWAP70* | 2588.521 | -0.273 | 0.0007 | 0.0131 |
| *CLTC* | 15454.767 | -0.273 | 0.0008 | 0.0153 |
| *SLC39A9* | 3289.906 | -0.273 | 0.0004 | 0.0096 |
| *CTDSPL2* | 1333.133 | -0.273 | 0.0004 | 0.0097 |
| *MEGF9* | 1875.131 | -0.273 | 0.0002 | 0.0061 |
| *SLC26A2* | 2169.443 | -0.272 | 0.0009 | 0.0162 |
| *TRIP11* | 2254.617 | -0.272 | 0.0002 | 0.0055 |
| *RAP2A* | 954.422 | -0.272 | 0.0029 | 0.0340 |
| *HIF1A* | 7195.077 | -0.271 | 0.0011 | 0.0184 |
| *DUSP3* | 1465.673 | -0.271 | 0.0011 | 0.0183 |
| *GLI3* | 848.207 | -0.271 | 0.0017 | 0.0239 |
| *ERC1* | 1478.445 | -0.271 | 0.0011 | 0.0182 |
| *BCAS1* | 2329.525 | -0.271 | 0.0002 | 0.0064 |
| *SASH1* | 1171.131 | -0.271 | 0.0021 | 0.0278 |
| *GNS* | 4904.471 | -0.271 | 0.0015 | 0.0225 |
| *NAA50* | 4808.539 | -0.270 | 0.0008 | 0.0145 |
| *CTNNB1* | 12903.511 | -0.270 | 0.0001 | 0.0049 |
| *MET* | 8789.866 | -0.270 | 0.0008 | 0.0154 |
| *SGPP2* | 5092.579 | -0.270 | 0.0019 | 0.0261 |
| *PLEKHF2* | 1906.199 | -0.270 | 0.0006 | 0.0118 |
| *NYNRIN* | 4534.058 | -0.269 | 0.0004 | 0.0102 |
| *ICK* | 2430.615 | -0.269 | 0.0002 | 0.0054 |
| *NCOA4* | 16733.476 | -0.269 | 0.0003 | 0.0086 |
| *TENM4* | 1395.963 | -0.268 | 0.0026 | 0.0318 |
| *UBXN7* | 634.004 | -0.268 | 0.0056 | 0.0496 |
| *GFPT1* | 4230.162 | -0.268 | 0.0037 | 0.0388 |
| *CTDSP2* | 6002.126 | -0.268 | 2.38E-05 | 0.0016 |
| *ZFP91* | 2853.391 | -0.267 | 0.0003 | 0.0085 |
| *MYO1D* | 5027.700 | -0.267 | 0.0004 | 0.0100 |
| *CHM* | 925.393 | -0.267 | 0.0034 | 0.0370 |
| *AHR* | 10432.852 | -0.267 | 0.0002 | 0.0058 |
| *MICAL2* | 1431.025 | -0.267 | 0.0036 | 0.0380 |
| *ACSL3* | 7922.880 | -0.266 | 0.0008 | 0.0147 |
| *RNF19B* | 1665.732 | -0.266 | 0.0030 | 0.0348 |
| *RAB31* | 1842.095 | -0.266 | 0.0008 | 0.0146 |
| *LONP2* | 2743.016 | -0.265 | 0.0005 | 0.0110 |
| *TSPYL1* | 5167.911 | -0.265 | 0.0005 | 0.0112 |
| *KIF1B* | 2002.505 | -0.265 | 0.0009 | 0.0161 |
| *EIF2AK2* | 873.675 | -0.265 | 0.0028 | 0.0331 |
| *XPR1* | 2215.702 | -0.265 | 0.0008 | 0.0154 |
| *DR1* | 1591.998 | -0.264 | 0.0008 | 0.0145 |
| *CSDE1* | 20438.923 | -0.264 | 0.0002 | 0.0068 |
| *RRM2B* | 1809.872 | -0.264 | 0.0023 | 0.0295 |
| *HINT3* | 1758.200 | -0.264 | 0.0047 | 0.0443 |
| *BAG5* | 2090.985 | -0.263 | 0.0003 | 0.0091 |
| *PCYOX1* | 3369.603 | -0.263 | 0.0004 | 0.0092 |
| *RHBDD1* | 971.459 | -0.263 | 0.0045 | 0.0429 |
| *ANTXR2* | 1133.487 | -0.263 | 0.0025 | 0.0307 |
| *BACH1* | 1281.538 | -0.263 | 0.0010 | 0.0164 |
| *TMEM2* | 1258.295 | -0.263 | 0.0024 | 0.0301 |
| *CANX* | 24724.295 | -0.262 | 0.0013 | 0.0198 |
| *DSG3* | 2180.317 | -0.262 | 0.0005 | 0.0114 |
| *USP6NL* | 1119.368 | -0.262 | 0.0026 | 0.0320 |
| *ERMP1* | 4374.172 | -0.262 | 0.0009 | 0.0160 |
| *LRRC8B* | 2117.408 | -0.261 | 0.0009 | 0.0156 |
| *CCNI* | 7229.168 | -0.261 | 0.0005 | 0.0108 |
| *TRIB1* | 1436.711 | -0.261 | 0.0036 | 0.0379 |
| *PTPN11* | 4095.200 | -0.261 | 0.0012 | 0.0184 |
| *PDP1* | 2289.354 | -0.261 | 0.0011 | 0.0178 |
| *GALNT2* | 5909.138 | -0.261 | 0.0002 | 0.0057 |
| *SHROOM3* | 3306.534 | -0.261 | 0.0027 | 0.0327 |
| *ECE1* | 12589.648 | -0.260 | 5.01E-05 | 0.0025 |
| *SIPA1L2* | 1594.144 | -0.260 | 0.0045 | 0.0435 |
| *DUSP16* | 1156.420 | -0.260 | 0.0034 | 0.0371 |
| *MICALL1* | 3473.563 | -0.259 | 0.0005 | 0.0108 |
| *RBBP5* | 800.633 | -0.259 | 0.0043 | 0.0419 |
| *SHROOM2* | 1320.859 | -0.259 | 0.0017 | 0.0247 |
| *LINC00657* | 16379.251 | -0.259 | 0.0002 | 0.0057 |
| *KAT6A* | 2429.400 | -0.259 | 0.0005 | 0.0106 |
| *CTTNBP2NL* | 2120.949 | -0.258 | 0.0031 | 0.0349 |
| *GPR56* | 8484.717 | -0.258 | 4.29E-05 | 0.0023 |
| *LDOC1L* | 1324.272 | -0.258 | 0.0040 | 0.0403 |
| *XRN2* | 3050.879 | -0.258 | 0.0011 | 0.0176 |
| *ZMPSTE24* | 3048.991 | -0.258 | 0.0012 | 0.0184 |
| *ZNF750* | 1033.609 | -0.257 | 0.0023 | 0.0295 |
| *LARS2* | 879.931 | -0.257 | 0.0029 | 0.0339 |
| *SGMS2* | 2845.658 | -0.257 | 0.0024 | 0.0299 |
| *LPHN2* | 1339.695 | -0.256 | 0.0016 | 0.0234 |
| *CNST* | 922.978 | -0.256 | 0.0051 | 0.0468 |
| *NDUFS1* | 2777.977 | -0.256 | 0.0014 | 0.0217 |
| *AMD1* | 3420.077 | -0.256 | 0.0053 | 0.0480 |
| *LYPLA1* | 2910.148 | -0.255 | 0.0006 | 0.0130 |
| *VPS18* | 1861.609 | -0.255 | 0.0046 | 0.0440 |
| *UPK1B* | 6260.472 | -0.255 | 0.0035 | 0.0376 |
| *PTBP3* | 5148.099 | -0.254 | 0.0011 | 0.0181 |
| *ANKS1A* | 1630.397 | -0.254 | 0.0028 | 0.0333 |
| *MITF* | 1258.034 | -0.254 | 0.0046 | 0.0441 |
| *ADAR* | 8018.643 | -0.254 | 0.0010 | 0.0166 |
| *GNAI3* | 2984.307 | -0.253 | 0.0030 | 0.0346 |
| *C1orf106* | 3893.263 | -0.253 | 0.0004 | 0.0102 |
| *LNX2* | 1278.094 | -0.253 | 0.0011 | 0.0180 |
| *CSNK2A1* | 3000.743 | -0.252 | 0.0005 | 0.0105 |
| *NDST1* | 3767.494 | -0.252 | 0.0002 | 0.0064 |
| *TMEM229B* | 898.993 | -0.252 | 0.0050 | 0.0459 |
| *ANKH* | 1878.815 | -0.252 | 0.0033 | 0.0360 |
| *FSTL1* | 4337.849 | -0.252 | 0.0027 | 0.0322 |
| *UHRF1BP1* | 1064.366 | -0.251 | 0.0032 | 0.0355 |
| *NCKAP1* | 7092.697 | -0.251 | 0.0004 | 0.0103 |
| *UBXN2B* | 1150.143 | -0.251 | 0.0035 | 0.0372 |
| *TMEM131* | 4334.100 | -0.251 | 0.0004 | 0.0101 |
| *EFCAB14* | 4837.432 | -0.251 | 0.0008 | 0.0154 |
| *SEC24A* | 1409.846 | -0.251 | 0.0032 | 0.0355 |
| *DSG2* | 8907.793 | -0.250 | 0.0018 | 0.0251 |
| *FAM168B* | 4390.347 | -0.250 | 0.0010 | 0.0167 |
| *HIPK2* | 3499.540 | -0.250 | 0.0002 | 0.0069 |
| *SESN2* | 1092.924 | -0.250 | 0.0039 | 0.0396 |
| *SYK* | 1998.139 | -0.249 | 0.0013 | 0.0205 |
| *TTLL12* | 2635.246 | -0.249 | 0.0029 | 0.0338 |
| *PTGFRN* | 4506.167 | -0.249 | 0.0004 | 0.0091 |
| *VCL* | 6216.308 | -0.249 | 0.0006 | 0.0130 |
| *NSD1* | 3256.080 | -0.249 | 0.0004 | 0.0099 |
| *SAMD12* | 1974.469 | -0.249 | 0.0024 | 0.0302 |
| *JAK1* | 5622.098 | -0.249 | 0.0026 | 0.0316 |
| *TANC2* | 2614.652 | -0.249 | 0.0006 | 0.0118 |
| *SPRED2* | 834.132 | -0.249 | 0.0040 | 0.0407 |
| *PAK2* | 3473.686 | -0.249 | 0.0004 | 0.0102 |
| *NFE2L1* | 10360.324 | -0.248 | 0.0039 | 0.0400 |
| *ZNF148* | 1629.793 | -0.248 | 0.0020 | 0.0273 |
| *ASXL2* | 1872.928 | -0.248 | 0.0022 | 0.0287 |
| *IPO5* | 4271.002 | -0.248 | 0.0019 | 0.0261 |
| *IFFO2* | 1583.370 | -0.248 | 0.0017 | 0.0247 |
| *NUP133* | 1388.833 | -0.248 | 0.0055 | 0.0488 |
| *CPT2* | 1471.114 | -0.248 | 0.0014 | 0.0209 |
| *C15orf39* | 1506.213 | -0.248 | 0.0045 | 0.0435 |
| *GTF2A1* | 1688.489 | -0.248 | 0.0020 | 0.0273 |
| *ST6GAL1* | 9731.200 | -0.248 | 0.0004 | 0.0102 |
| *SLC38A2* | 19137.621 | -0.248 | 1.74E-05 | 0.0013 |
| *ABHD17C* | 2097.179 | -0.247 | 0.0026 | 0.0320 |
| *CRK* | 3067.281 | -0.247 | 0.0004 | 0.0100 |
| *IPO8* | 1967.149 | -0.247 | 0.0019 | 0.0258 |
| *CHSY1* | 1075.348 | -0.247 | 0.0049 | 0.0457 |
| *CHST3* | 1182.905 | -0.247 | 0.0054 | 0.0486 |
| *CDC73* | 1663.982 | -0.246 | 0.0032 | 0.0357 |
| *ZNF217* | 3354.582 | -0.246 | 0.0003 | 0.0076 |
| *TMEM127* | 3301.571 | -0.246 | 0.0006 | 0.0123 |
| *NEK7* | 1808.375 | -0.246 | 0.0031 | 0.0353 |
| *NUP153* | 1966.557 | -0.246 | 0.0023 | 0.0292 |
| *SH3BP4* | 2984.085 | -0.245 | 0.0020 | 0.0266 |
| *RAB11FIP1* | 8445.198 | -0.245 | 0.0023 | 0.0291 |
| *AREL1* | 2055.961 | -0.245 | 0.0006 | 0.0117 |
| *LRPPRC* | 5274.593 | -0.244 | 0.0013 | 0.0203 |
| *HYOU1* | 5985.691 | -0.244 | 0.0041 | 0.0412 |
| *YME1L1* | 5788.221 | -0.244 | 0.0030 | 0.0348 |
| *VPS4B* | 3944.665 | -0.244 | 0.0032 | 0.0355 |
| *ARID5B* | 2068.724 | -0.244 | 0.0020 | 0.0269 |
| *CCDC47* | 4306.887 | -0.244 | 0.0022 | 0.0288 |
| *CDV3* | 3952.615 | -0.244 | 0.0014 | 0.0212 |
| *ARCN1* | 5815.403 | -0.243 | 0.0009 | 0.0158 |
| *APLP2* | 34170.078 | -0.243 | 0.0004 | 0.0096 |
| *TNPO1* | 3950.246 | -0.243 | 0.0005 | 0.0108 |
| *TRIP12* | 7752.814 | -0.243 | 0.0026 | 0.0313 |
| *MAN1A2* | 3863.749 | -0.242 | 0.0011 | 0.0176 |
| *CHURC1* | 1650.979 | -0.242 | 0.0048 | 0.0447 |
| *AGPS* | 2415.678 | -0.242 | 0.0054 | 0.0485 |
| *BCL2L11* | 1111.271 | -0.242 | 0.0032 | 0.0357 |
| *ELF1* | 3394.525 | -0.242 | 0.0011 | 0.0176 |
| *ATP8B1* | 5388.086 | -0.241 | 0.0015 | 0.0227 |
| *MKL2* | 4346.255 | -0.241 | 0.0003 | 0.0088 |
| *IARS2* | 4475.940 | -0.241 | 0.0016 | 0.0228 |
| *PPP1CB* | 10208.328 | -0.241 | 0.0011 | 0.0180 |
| *NCOA2* | 1396.482 | -0.241 | 0.0024 | 0.0298 |
| *DCAF12* | 2716.503 | -0.241 | 0.0027 | 0.0322 |
| *VANGL1* | 2110.030 | -0.241 | 0.0016 | 0.0231 |
| *ADCK2* | 861.613 | -0.240 | 0.0050 | 0.0458 |
| *GSK3B* | 2758.378 | -0.240 | 0.0008 | 0.0145 |
| *DIP2B* | 2005.157 | -0.240 | 0.0014 | 0.0217 |
| *RBM27* | 1695.937 | -0.240 | 0.0013 | 0.0201 |
| *CAMSAP1* | 2565.183 | -0.239 | 0.0035 | 0.0372 |
| *CLSTN1* | 17727.487 | -0.239 | 3.44E-05 | 0.0020 |
| *ARPP19* | 5578.135 | -0.239 | 0.0004 | 0.0100 |
| *SLC30A9* | 2844.344 | -0.239 | 0.0033 | 0.0360 |
| *TP63* | 5758.151 | -0.239 | 0.0011 | 0.0176 |
| *SRPK1* | 3334.272 | -0.239 | 0.0019 | 0.0262 |
| *SSFA2* | 3999.761 | -0.239 | 0.0008 | 0.0146 |
| *TLE1* | 1458.626 | -0.238 | 0.0050 | 0.0461 |
| *SDR16C5* | 1544.360 | -0.238 | 0.0045 | 0.0434 |
| *CPSF2* | 2432.929 | -0.238 | 0.0040 | 0.0401 |
| *IQGAP1* | 9668.235 | -0.238 | 0.0004 | 0.0097 |
| *MARCKS* | 10801.590 | -0.238 | 0.0015 | 0.0227 |
| *IPO7* | 5489.522 | -0.238 | 0.0018 | 0.0258 |
| *COG5* | 1860.909 | -0.238 | 0.0043 | 0.0423 |
| *FEM1A* | 1607.391 | -0.238 | 0.0036 | 0.0383 |
| *EDEM1* | 2484.227 | -0.238 | 0.0027 | 0.0322 |
| *FBXL3* | 1436.619 | -0.237 | 0.0021 | 0.0276 |
| *AGAP1* | 1982.645 | -0.237 | 0.0037 | 0.0384 |
| *BTG1* | 5815.574 | -0.237 | 0.0005 | 0.0109 |
| *PHF3* | 2718.583 | -0.237 | 0.0015 | 0.0225 |
| *ATG13* | 2276.235 | -0.237 | 0.0039 | 0.0400 |
| *SMARCC1* | 5516.870 | -0.237 | 0.0018 | 0.0252 |
| *ACBD5* | 1483.689 | -0.236 | 0.0032 | 0.0357 |
| *ARNT* | 1634.089 | -0.236 | 0.0022 | 0.0285 |
| *AFF4* | 4703.524 | -0.236 | 0.0012 | 0.0187 |
| *LRRC8A* | 7263.329 | -0.236 | 0.0015 | 0.0228 |
| *OPN3* | 932.947 | -0.235 | 0.0055 | 0.0491 |
| *TBC1D16* | 2229.259 | -0.235 | 0.0010 | 0.0169 |
| *ZBTB41* | 1158.496 | -0.235 | 0.0048 | 0.0447 |
| *CASK* | 2478.356 | -0.235 | 0.0012 | 0.0184 |
| *PLS1* | 3500.880 | -0.235 | 0.0039 | 0.0396 |
| *GPD2* | 2768.317 | -0.234 | 0.0021 | 0.0278 |
| *RBM47* | 5674.462 | -0.234 | 0.0012 | 0.0194 |
| *PSME4* | 4989.734 | -0.234 | 0.0012 | 0.0193 |
| *UBE2G1* | 2488.092 | -0.234 | 0.0015 | 0.0218 |
| *RIN2* | 3304.038 | -0.234 | 0.0012 | 0.0193 |
| *PARP1* | 4287.757 | -0.234 | 0.0020 | 0.0273 |
| *SENP2* | 1087.888 | -0.234 | 0.0033 | 0.0360 |
| *QSER1* | 1965.988 | -0.234 | 0.0039 | 0.0400 |
| *USP1* | 1645.873 | -0.234 | 0.0044 | 0.0427 |
| *WLS* | 2114.847 | -0.233 | 0.0012 | 0.0193 |
| *SIK3* | 1213.381 | -0.233 | 0.0034 | 0.0370 |
| *PAX9* | 2675.021 | -0.233 | 0.0011 | 0.0176 |
| *MAN2A1* | 1753.379 | -0.233 | 0.0051 | 0.0469 |
| *RALA* | 1668.045 | -0.232 | 0.0028 | 0.0333 |
| *DDX3X* | 12385.927 | -0.232 | 0.0002 | 0.0071 |
| *TTC9* | 3571.542 | -0.232 | 0.0021 | 0.0277 |
| *ATP5A1* | 18214.503 | -0.232 | 0.0009 | 0.0160 |
| *UBR3* | 1599.768 | -0.231 | 0.0039 | 0.0396 |
| *SLC35B2* | 1998.402 | -0.231 | 0.0019 | 0.0265 |
| *CKAP4* | 5141.715 | -0.231 | 0.0037 | 0.0390 |
| *NNT* | 2752.901 | -0.231 | 0.0016 | 0.0228 |
| *CAPZA2* | 3424.137 | -0.231 | 0.0032 | 0.0355 |
| *WAPAL* | 2380.830 | -0.231 | 0.0040 | 0.0404 |
| *SLC43A3* | 1906.858 | -0.231 | 0.0036 | 0.0379 |
| *MFSD6* | 4661.020 | -0.230 | 0.0036 | 0.0381 |
| *LRRC16A* | 2951.495 | -0.230 | 0.0015 | 0.0222 |
| *USP22* | 9749.231 | -0.230 | 0.0001 | 0.0045 |
| *C1orf198* | 1518.856 | -0.230 | 0.0024 | 0.0304 |
| *CHST9* | 5353.510 | -0.229 | 0.0032 | 0.0355 |
| *MAL2* | 11537.713 | -0.229 | 0.0026 | 0.0316 |
| *SEC24B* | 1904.322 | -0.228 | 0.0027 | 0.0324 |
| *TLK1* | 2419.363 | -0.228 | 0.0041 | 0.0412 |
| *DCUN1D1* | 1537.281 | -0.228 | 0.0046 | 0.0437 |
| *DCTN5* | 2430.683 | -0.228 | 0.0032 | 0.0355 |
| *SIPA1L3* | 2538.306 | -0.228 | 0.0023 | 0.0292 |
| *CAPRIN1* | 10035.681 | -0.228 | 0.0029 | 0.0338 |
| *PSD3* | 2928.781 | -0.228 | 0.0013 | 0.0201 |
| *POM121C* | 1287.774 | -0.228 | 0.0051 | 0.0470 |
| *SIN3A* | 3360.823 | -0.228 | 0.0038 | 0.0393 |
| *CX3CL1* | 5665.770 | -0.228 | 0.0052 | 0.0474 |
| *SEC23IP* | 2282.325 | -0.227 | 0.0045 | 0.0431 |
| *KIAA0100* | 6193.403 | -0.227 | 0.0054 | 0.0485 |
| *IRF2BPL* | 2948.370 | -0.227 | 0.0054 | 0.0486 |
| *ZFR* | 2847.487 | -0.227 | 0.0018 | 0.0250 |
| *GLCE* | 1153.112 | -0.227 | 0.0048 | 0.0451 |
| *IFNAR1* | 3127.940 | -0.226 | 0.0053 | 0.0481 |
| *CPEB4* | 2145.539 | -0.226 | 0.0027 | 0.0327 |
| *FYTTD1* | 2216.377 | -0.226 | 0.0022 | 0.0284 |
| *FGFR2* | 4288.143 | -0.225 | 0.0006 | 0.0118 |
| *POFUT1* | 1825.307 | -0.225 | 0.0033 | 0.0364 |
| *TM9SF3* | 10618.954 | -0.225 | 0.0020 | 0.0274 |
| *RAB14* | 5896.997 | -0.225 | 0.0039 | 0.0398 |
| *PARVA* | 3693.139 | -0.225 | 0.0020 | 0.0273 |
| *OXR1* | 2216.087 | -0.224 | 0.0020 | 0.0269 |
| *PRKDC* | 5479.720 | -0.224 | 0.0023 | 0.0292 |
| *PRKAR1A* | 11884.985 | -0.224 | 0.0026 | 0.0316 |
| *CDC42SE2* | 2575.828 | -0.223 | 0.0055 | 0.0490 |
| *TCF12* | 2278.308 | -0.223 | 0.0028 | 0.0334 |
| *CASC3* | 2608.936 | -0.223 | 0.0014 | 0.0212 |
| *SESN3* | 2180.075 | -0.223 | 0.0038 | 0.0396 |
| *TMED10* | 10017.074 | -0.223 | 0.0043 | 0.0419 |
| *FEM1B* | 3092.337 | -0.223 | 0.0019 | 0.0265 |
| *MTDH* | 5783.530 | -0.222 | 0.0019 | 0.0263 |
| *CCDC6* | 4935.369 | -0.222 | 0.0010 | 0.0166 |
| *HBP1* | 2296.835 | -0.222 | 0.0054 | 0.0483 |
| *EIF3A* | 10859.430 | -0.222 | 0.0032 | 0.0357 |
| *CDK6* | 3328.779 | -0.222 | 0.0054 | 0.0483 |
| *KIAA1161* | 2434.208 | -0.221 | 0.0033 | 0.0362 |
| *FAM84B* | 2272.445 | -0.221 | 0.0037 | 0.0388 |
| *APP* | 31903.329 | -0.221 | 0.0020 | 0.0274 |
| *ZNF609* | 4189.495 | -0.221 | 0.0017 | 0.0248 |
| *TMTC3* | 2507.010 | -0.221 | 0.0023 | 0.0295 |
| *OAT* | 9051.704 | -0.220 | 0.0044 | 0.0426 |
| *PRPF8* | 10036.591 | -0.220 | 0.0035 | 0.0375 |
| *MAML1* | 1970.086 | -0.220 | 0.0023 | 0.0292 |
| *GSR* | 5991.473 | -0.220 | 0.0014 | 0.0217 |
| *KDM6A* | 1731.703 | -0.220 | 0.0036 | 0.0380 |
| *NOTCH2* | 5906.442 | -0.219 | 0.0041 | 0.0409 |
| *NCOA6* | 2737.562 | -0.219 | 0.0029 | 0.0336 |
| *MON1B* | 1639.754 | -0.219 | 0.0043 | 0.0419 |
| *ANKRD50* | 2359.867 | -0.219 | 0.0032 | 0.0355 |
| *KREMEN1* | 3273.315 | -0.219 | 0.0045 | 0.0433 |
| *UBE3C* | 3393.801 | -0.219 | 0.0054 | 0.0484 |
| *TNKS2* | 3742.092 | -0.218 | 0.0018 | 0.0252 |
| *UBFD1* | 2344.487 | -0.218 | 0.0033 | 0.0361 |
| *UBE2H* | 9018.466 | -0.218 | 0.0007 | 0.0132 |
| *RAB6A* | 5650.634 | -0.218 | 0.0019 | 0.0259 |
| *CDKN1A* | 9105.240 | -0.218 | 0.0013 | 0.0198 |
| *ATP2A2* | 20326.361 | -0.218 | 0.0016 | 0.0228 |
| *NFKB1* | 3494.913 | -0.218 | 0.0049 | 0.0456 |
| *DDB1* | 10087.330 | -0.217 | 0.0049 | 0.0454 |
| *ATP11B* | 2853.980 | -0.217 | 0.0031 | 0.0350 |
| *SMARCA5* | 5349.578 | -0.217 | 0.0028 | 0.0333 |
| *PBRM1* | 2776.416 | -0.217 | 0.0041 | 0.0410 |
| *HMGA1* | 9544.643 | -0.217 | 0.0008 | 0.0144 |
| *LMTK2* | 2682.174 | -0.217 | 0.0021 | 0.0279 |
| *JMY* | 1621.932 | -0.216 | 0.0050 | 0.0458 |
| *WNK1* | 5210.487 | -0.216 | 0.0006 | 0.0118 |
| *SP3* | 4190.786 | -0.216 | 0.0016 | 0.0231 |
| *MR1* | 1562.781 | -0.215 | 0.0048 | 0.0449 |
| *BRPF3* | 2125.430 | -0.215 | 0.0031 | 0.0353 |
| *ACAP2* | 1950.935 | -0.215 | 0.0029 | 0.0338 |
| *LGR4* | 3706.169 | -0.215 | 0.0013 | 0.0199 |
| *ABLIM1* | 9974.526 | -0.215 | 0.0003 | 0.0086 |
| *SMAD5* | 2945.144 | -0.215 | 0.0037 | 0.0389 |
| *MED13* | 2593.075 | -0.215 | 0.0025 | 0.0306 |
| *ZMAT3* | 3171.359 | -0.214 | 0.0026 | 0.0318 |
| *LAMC1* | 4182.560 | -0.214 | 0.0040 | 0.0404 |
| *TNKS* | 2036.409 | -0.214 | 0.0039 | 0.0400 |
| *HECTD1* | 8030.409 | -0.214 | 0.0008 | 0.0144 |
| *KIAA0232* | 2442.264 | -0.214 | 0.0042 | 0.0417 |
| *NUDT4* | 2839.484 | -0.214 | 0.0050 | 0.0463 |
| *SCARB2* | 6169.373 | -0.214 | 0.0032 | 0.0355 |
| *CYFIP1* | 4894.892 | -0.213 | 0.0033 | 0.0361 |
| *PDPK1* | 2303.072 | -0.213 | 0.0034 | 0.0369 |
| *MMP14* | 13643.826 | -0.213 | 0.0021 | 0.0276 |
| *FAM60A* | 2706.905 | -0.213 | 0.0025 | 0.0310 |
| *KDM5B* | 5417.101 | -0.212 | 0.0010 | 0.0169 |
| *ZDHHC20* | 4480.110 | -0.212 | 0.0036 | 0.0381 |
| *FURIN* | 3062.369 | -0.211 | 0.0052 | 0.0471 |
| *DNAJC13* | 2181.771 | -0.211 | 0.0026 | 0.0316 |
| *C16orf62* | 1649.577 | -0.211 | 0.0041 | 0.0407 |
| *STARD7* | 5072.265 | -0.211 | 0.0054 | 0.0484 |
| *RNF152* | 1476.375 | -0.211 | 0.0051 | 0.0469 |
| *SF3A1* | 4238.335 | -0.210 | 0.0042 | 0.0412 |
| *PAFAH1B1* | 5871.954 | -0.210 | 0.0031 | 0.0353 |
| *API5* | 4599.270 | -0.210 | 0.0052 | 0.0473 |
| *EEF2* | 76001.382 | -0.210 | 0.0006 | 0.0117 |
| *YTHDF3* | 3096.779 | -0.209 | 0.0054 | 0.0484 |
| *ZNF644* | 2089.862 | -0.209 | 0.0037 | 0.0389 |
| *KIF13B* | 3746.144 | -0.209 | 0.0045 | 0.0432 |
| *SEPHS2* | 2285.718 | -0.208 | 0.0043 | 0.0419 |
| *NCOA3* | 3410.508 | -0.208 | 0.0033 | 0.0362 |
| *TGOLN2* | 6792.696 | -0.208 | 0.0026 | 0.0314 |
| *EEF1A1* | 108491.688 | -0.207 | 0.0030 | 0.0344 |
| *FAM120A* | 8105.513 | -0.207 | 0.0035 | 0.0376 |
| *FZD6* | 5084.703 | -0.207 | 0.0015 | 0.0222 |
| *RPS6KA3* | 4042.996 | -0.206 | 0.0031 | 0.0353 |
| *GPRC5A* | 4167.146 | -0.206 | 0.0048 | 0.0450 |
| *ZDHHC5* | 5143.539 | -0.206 | 0.0042 | 0.0417 |
| *EXT2* | 2215.527 | -0.206 | 0.0054 | 0.0485 |
| *LAMP1* | 9260.882 | -0.205 | 0.0035 | 0.0376 |
| *SRP72* | 3365.679 | -0.205 | 0.0049 | 0.0457 |
| *GIGYF2* | 2698.315 | -0.205 | 0.0052 | 0.0471 |
| *SLC38A1* | 9030.787 | -0.204 | 0.0023 | 0.0292 |
| *PARD3* | 3005.219 | -0.204 | 0.0056 | 0.0497 |
| *NF1* | 3070.896 | -0.203 | 0.0027 | 0.0324 |
| *HCFC1* | 3675.431 | -0.202 | 0.0057 | 0.0498 |
| *F11R* | 13530.840 | -0.202 | 0.0019 | 0.0265 |
| *PAM* | 4647.121 | -0.202 | 0.0043 | 0.0423 |
| *CREBBP* | 2899.346 | -0.201 | 0.0046 | 0.0436 |
| *ARID1B* | 3831.436 | -0.201 | 0.0021 | 0.0277 |
| *SORL1* | 2960.008 | -0.201 | 0.0043 | 0.0419 |
| *ARHGEF12* | 5907.050 | -0.200 | 0.0027 | 0.0321 |
| *TAOK1* | 3213.154 | -0.200 | 0.0032 | 0.0357 |
| *EIF4EBP2* | 6558.960 | -0.199 | 0.0042 | 0.0412 |
| *PALLD* | 3592.944 | -0.198 | 0.0052 | 0.0472 |
| *YAP1* | 6063.516 | -0.197 | 0.0025 | 0.0306 |
| *HADHA* | 8147.188 | -0.196 | 0.0048 | 0.0449 |
| *KIF5B* | 9975.350 | -0.195 | 0.0034 | 0.0366 |
| *DNAJC5* | 6437.608 | -0.194 | 0.0039 | 0.0401 |
| *AQP3* | 37886.960 | -0.194 | 0.0049 | 0.0456 |
| *RAB3GAP1* | 2646.804 | -0.193 | 0.0052 | 0.0473 |
| *CORO2A* | 4806.880 | -0.193 | 0.0054 | 0.0483 |
| *MARK2* | 3408.432 | -0.192 | 0.0041 | 0.0409 |
| *FNBP1L* | 3676.804 | -0.191 | 0.0048 | 0.0449 |
| *ADNP* | 5425.829 | -0.190 | 0.0022 | 0.0284 |
| *EIF3L* | 9896.262 | -0.190 | 0.0036 | 0.0383 |
| *GLG1* | 6138.673 | -0.190 | 0.0029 | 0.0338 |
| *WDR82* | 4487.839 | -0.189 | 0.0044 | 0.0427 |
| *GM2A* | 9290.589 | -0.185 | 0.0048 | 0.0451 |
| *HNRNPA0* | 6574.560 | -0.182 | 0.0031 | 0.0351 |
| *EIF4H* | 7044.971 | -0.182 | 0.0057 | 0.0498 |
| *EIF4G2* | 32460.263 | -0.180 | 0.0056 | 0.0496 |
| *HNRNPUL1* | 11927.357 | -0.176 | 0.0042 | 0.0417 |
| *RUNX1* | 7530.859 | -0.170 | 0.0057 | 0.0499 |
| *RAB13* | 3134.501 | 0.185 | 0.0054 | 0.0483 |
| *NASP* | 3407.043 | 0.195 | 0.0031 | 0.0351 |
| *SLC9A3R2* | 5080.328 | 0.196 | 0.0029 | 0.0338 |
| *RARRES3* | 3309.530 | 0.198 | 0.0053 | 0.0477 |
| *TRIM22* | 7020.473 | 0.199 | 0.0042 | 0.0417 |
| *MGST2* | 1992.803 | 0.204 | 0.0048 | 0.0448 |
| *SSBP4* | 2793.836 | 0.204 | 0.0042 | 0.0415 |
| *ERCC1* | 3071.745 | 0.209 | 0.0021 | 0.0276 |
| *SGSM3* | 6018.405 | 0.209 | 0.0017 | 0.0242 |
| *MRPS21* | 1920.725 | 0.210 | 0.0056 | 0.0493 |
| *PHIP* | 6560.445 | 0.214 | 0.0043 | 0.0420 |
| *SERF2* | 4395.427 | 0.214 | 0.0030 | 0.0344 |
| *PNPLA2* | 6733.444 | 0.216 | 0.0035 | 0.0372 |
| *JAG2* | 6828.045 | 0.216 | 0.0047 | 0.0444 |
| *DOCK5* | 3558.819 | 0.221 | 0.0032 | 0.0355 |
| *HMG20B* | 4681.984 | 0.222 | 0.0034 | 0.0370 |
| *CIB1* | 6071.087 | 0.222 | 0.0008 | 0.0146 |
| *FUS* | 7961.905 | 0.223 | 0.0010 | 0.0164 |
| *SPG7* | 3287.918 | 0.224 | 0.0038 | 0.0395 |
| *TOMM6* | 2581.134 | 0.225 | 0.0041 | 0.0409 |
| *CMAHP* | 3250.241 | 0.228 | 0.0012 | 0.0184 |
| *ATPIF1* | 3828.818 | 0.228 | 0.0008 | 0.0144 |
| *IFT80* | 2648.937 | 0.229 | 0.0009 | 0.0160 |
| *IKBKB* | 2803.799 | 0.230 | 0.0031 | 0.0353 |
| *PRMT7* | 1064.561 | 0.231 | 0.0055 | 0.0490 |
| *FAM111A* | 2809.725 | 0.231 | 0.0039 | 0.0396 |
| *PPP1R16A* | 2976.565 | 0.232 | 0.0023 | 0.0289 |
| *NPDC1* | 2653.636 | 0.232 | 0.0034 | 0.0369 |
| *FAN1* | 1556.439 | 0.232 | 0.0020 | 0.0273 |
| *TIMP1* | 3211.859 | 0.233 | 0.0007 | 0.0133 |
| *MYO19* | 2503.571 | 0.234 | 0.0050 | 0.0463 |
| *PDCD5* | 1173.198 | 0.234 | 0.0039 | 0.0396 |
| *PTTG1* | 1058.726 | 0.235 | 0.0057 | 0.0498 |
| *SH3BP2* | 4578.491 | 0.239 | 0.0048 | 0.0448 |
| *SFPQ* | 15962.310 | 0.240 | 0.0005 | 0.0117 |
| *CEP164* | 1319.377 | 0.241 | 0.0047 | 0.0446 |
| *IFT172* | 2253.864 | 0.241 | 0.0010 | 0.0164 |
| *NFATC2IP* | 2423.330 | 0.242 | 0.0045 | 0.0433 |
| *TARBP1* | 1610.875 | 0.242 | 0.0053 | 0.0477 |
| *RBM39* | 10642.395 | 0.242 | 0.0036 | 0.0380 |
| *MTA1* | 3266.965 | 0.242 | 0.0044 | 0.0427 |
| *FAM160A2* | 1473.689 | 0.243 | 0.0047 | 0.0444 |
| *RALGDS* | 2791.997 | 0.244 | 0.0037 | 0.0389 |
| *COQ4* | 2363.252 | 0.245 | 0.0007 | 0.0141 |
| *SRSF6* | 9508.552 | 0.245 | 0.0017 | 0.0240 |
| *NADSYN1* | 2532.434 | 0.245 | 0.0006 | 0.0118 |
| *ANXA8* | 3207.896 | 0.245 | 0.0033 | 0.0364 |
| *CTBP1* | 5679.925 | 0.247 | 0.0026 | 0.0312 |
| *ZNF211* | 929.618 | 0.248 | 0.0044 | 0.0424 |
| *BCL6* | 12444.754 | 0.248 | 0.0026 | 0.0315 |
| *RUFY3* | 1658.434 | 0.249 | 0.0051 | 0.0467 |
| *ARL16* | 1291.775 | 0.249 | 0.0039 | 0.0401 |
| *VPS13A* | 2997.452 | 0.249 | 0.0019 | 0.0261 |
| *SZT2* | 1852.544 | 0.249 | 0.0038 | 0.0395 |
| *EPS8L2* | 9635.735 | 0.250 | 0.0017 | 0.0240 |
| *MRPS25* | 1870.039 | 0.250 | 0.0022 | 0.0285 |
| *POMT1* | 1264.038 | 0.251 | 0.0019 | 0.0259 |
| *ASPSCR1* | 863.057 | 0.252 | 0.0045 | 0.0431 |
| *CFI* | 2424.145 | 0.253 | 0.0017 | 0.0239 |
| *SHFM1* | 2182.923 | 0.255 | 0.0005 | 0.0106 |
| *N4BP2L2* | 2512.188 | 0.255 | 0.0016 | 0.0229 |
| *DDX39A* | 1464.343 | 0.255 | 0.0018 | 0.0251 |
| *PRPF38B* | 3376.339 | 0.257 | 0.0011 | 0.0176 |
| *ABCA7* | 4591.863 | 0.257 | 0.0045 | 0.0430 |
| *TRMT2A* | 1691.228 | 0.259 | 0.0022 | 0.0286 |
| *C1orf52* | 780.773 | 0.259 | 0.0037 | 0.0386 |
| *ODF2L* | 4923.751 | 0.259 | 0.0004 | 0.0098 |
| *WDR60* | 1929.501 | 0.259 | 0.0015 | 0.0228 |
| *HKR1* | 1479.873 | 0.259 | 0.0029 | 0.0337 |
| *SUGP2* | 5509.923 | 0.259 | 0.0010 | 0.0166 |
| *MAPRE3* | 1475.092 | 0.260 | 0.0009 | 0.0163 |
| *AKAP17A* | 2884.460 | 0.260 | 0.0034 | 0.0366 |
| *HDHD3* | 1479.008 | 0.261 | 0.0021 | 0.0275 |
| *PLEKHS1* | 26220.748 | 0.261 | 0.0004 | 0.0095 |
| *TRIP10* | 4460.082 | 0.261 | 0.0018 | 0.0250 |
| *CCHCR1* | 1320.929 | 0.262 | 0.0027 | 0.0323 |
| *DMKN* | 1690.614 | 0.262 | 0.0038 | 0.0395 |
| *TMEM120B* | 954.080 | 0.262 | 0.0037 | 0.0388 |
| *NOL12* | 689.529 | 0.262 | 0.0057 | 0.0498 |
| *POLR2I* | 781.045 | 0.264 | 0.0040 | 0.0403 |
| *SRSF7* | 4502.884 | 0.264 | 0.0001 | 0.0052 |
| *CAPN15* | 2573.520 | 0.264 | 0.0052 | 0.0474 |
| *PPOX* | 916.901 | 0.264 | 0.0032 | 0.0355 |
| *SIRT6* | 844.885 | 0.265 | 0.0043 | 0.0419 |
| *CNTNAP3* | 925.491 | 0.265 | 0.0035 | 0.0372 |
| *TBX1* | 1769.567 | 0.266 | 0.0055 | 0.0486 |
| *HMGN3* | 5420.954 | 0.266 | 0.0001 | 0.0044 |
| *NPRL2* | 710.352 | 0.267 | 0.0056 | 0.0495 |
| *FASTK* | 2793.249 | 0.267 | 0.0012 | 0.0192 |
| *LRCH4* | 4103.081 | 0.267 | 0.0022 | 0.0284 |
| *ANKRD13D* | 1801.944 | 0.268 | 0.0032 | 0.0356 |
| *C21orf59* | 3785.350 | 0.268 | 0.0005 | 0.0113 |
| *MRPL24* | 877.391 | 0.269 | 0.0046 | 0.0442 |
| *CEP95* | 1895.722 | 0.269 | 0.0033 | 0.0365 |
| *HCG18* | 917.963 | 0.269 | 0.0043 | 0.0420 |
| *AZI1* | 1395.603 | 0.270 | 0.0027 | 0.0325 |
| *SRA1* | 1162.295 | 0.270 | 0.0009 | 0.0158 |
| *LOC100862671* | 1086.529 | 0.270 | 0.0036 | 0.0378 |
| *OGT* | 7289.024 | 0.270 | 0.0054 | 0.0483 |
| *RBCK1* | 3813.276 | 0.271 | 0.0009 | 0.0160 |
| *SLC25A28* | 1578.984 | 0.272 | 0.0020 | 0.0271 |
| *AP4B1* | 775.995 | 0.273 | 0.0043 | 0.0421 |
| *HMGN5* | 665.041 | 0.273 | 0.0048 | 0.0451 |
| *ZNF738* | 708.765 | 0.273 | 0.0048 | 0.0450 |
| *ZMYND19* | 852.596 | 0.273 | 0.0054 | 0.0483 |
| *SREK1* | 4213.796 | 0.274 | 0.0011 | 0.0175 |
| *ZNF84* | 1649.281 | 0.274 | 0.0021 | 0.0275 |
| *FAM73B* | 1442.333 | 0.275 | 0.0053 | 0.0477 |
| *FANCL* | 911.505 | 0.275 | 0.0054 | 0.0486 |
| *TRMT13* | 872.985 | 0.276 | 0.0027 | 0.0322 |
| *SS18L1* | 1290.458 | 0.276 | 0.0019 | 0.0261 |
| *CAPN10* | 958.256 | 0.277 | 0.0048 | 0.0451 |
| *CRELD1* | 997.118 | 0.277 | 0.0016 | 0.0233 |
| *OBSL1* | 3086.782 | 0.277 | 0.0024 | 0.0299 |
| *IFT43* | 1261.299 | 0.278 | 0.0006 | 0.0130 |
| *ATG4B* | 2286.080 | 0.278 | 0.0017 | 0.0249 |
| *KRI1* | 1818.318 | 0.278 | 0.0008 | 0.0147 |
| *METTL3* | 1513.346 | 0.278 | 0.0011 | 0.0176 |
| *PLCG1* | 2648.234 | 0.279 | 0.0019 | 0.0259 |
| *C19orf60* | 927.264 | 0.279 | 0.0051 | 0.0468 |
| *OSER1* | 1625.185 | 0.279 | 0.0003 | 0.0083 |
| *SNRNP70* | 8000.431 | 0.279 | 0.0005 | 0.0107 |
| *PSME2* | 3512.056 | 0.279 | 2.33E-05 | 0.0016 |
| *CLU* | 6758.532 | 0.280 | 0.0034 | 0.0371 |
| *HEMK1* | 731.236 | 0.280 | 0.0054 | 0.0483 |
| *PNKP* | 1649.874 | 0.280 | 0.0022 | 0.0283 |
| *SIGIRR* | 1485.087 | 0.281 | 0.0011 | 0.0175 |
| *SRSF11* | 8611.636 | 0.281 | 0.0026 | 0.0313 |
| *FAM76B* | 1060.313 | 0.281 | 0.0055 | 0.0490 |
| *CSPP1* | 2065.315 | 0.282 | 0.0004 | 0.0104 |
| *SUSD4* | 1468.606 | 0.282 | 0.0005 | 0.0113 |
| *PAXBP1* | 1748.226 | 0.283 | 0.0024 | 0.0300 |
| *POFUT2* | 1304.334 | 0.284 | 0.0007 | 0.0136 |
| *ACADVL* | 22125.674 | 0.285 | 0.0002 | 0.0058 |
| *ARVCF* | 1600.189 | 0.285 | 0.0047 | 0.0443 |
| *TDRP* | 757.173 | 0.285 | 0.0028 | 0.0334 |
| *UPF3A* | 2152.081 | 0.285 | 0.0004 | 0.0097 |
| *EHBP1L1* | 6035.772 | 0.285 | 0.0021 | 0.0278 |
| *NEURL4* | 1539.547 | 0.286 | 0.0017 | 0.0242 |
| *ORMDL1* | 2304.058 | 0.286 | 0.0025 | 0.0310 |
| *ZNF204P* | 1017.303 | 0.286 | 0.0014 | 0.0215 |
| *CYTH2* | 8275.629 | 0.286 | 0.0006 | 0.0122 |
| *S100A13* | 1264.493 | 0.286 | 0.0004 | 0.0102 |
| *ZNF224* | 965.258 | 0.286 | 0.0057 | 0.0498 |
| *ARHGEF1* | 2242.886 | 0.287 | 0.0029 | 0.0340 |
| *TRPT1* | 1019.442 | 0.287 | 0.0044 | 0.0427 |
| *CCDC130* | 1333.042 | 0.287 | 0.0042 | 0.0417 |
| *BTN3A1* | 814.559 | 0.287 | 0.0028 | 0.0334 |
| *SDR39U1* | 1189.591 | 0.287 | 0.0048 | 0.0451 |
| *DDX56* | 2573.687 | 0.288 | 0.0011 | 0.0176 |
| *TJAP1* | 1693.041 | 0.288 | 0.0027 | 0.0323 |
| *SH2D3A* | 1827.462 | 0.288 | 0.0005 | 0.0111 |
| *POGZ* | 3271.387 | 0.288 | 0.0015 | 0.0228 |
| *ZBTB48* | 945.693 | 0.288 | 0.0051 | 0.0468 |
| *PARP10* | 2427.810 | 0.289 | 0.0006 | 0.0126 |
| *RBM25* | 6962.323 | 0.290 | 0.0002 | 0.0057 |
| *SULT1A1* | 761.067 | 0.290 | 0.0032 | 0.0358 |
| *HSPB11* | 1075.485 | 0.291 | 0.0004 | 0.0095 |
| *SPEF1* | 968.188 | 0.291 | 0.0054 | 0.0483 |
| *RGL2* | 3844.960 | 0.291 | 0.0011 | 0.0176 |
| *KIFC3* | 1256.816 | 0.292 | 0.0014 | 0.0215 |
| *ZNF248* | 869.147 | 0.292 | 0.0032 | 0.0356 |
| *ABCC3* | 2011.154 | 0.293 | 0.0013 | 0.0205 |
| *CCDC66* | 849.335 | 0.295 | 0.0046 | 0.0441 |
| *TNFAIP2* | 22141.605 | 0.295 | 0.0044 | 0.0425 |
| *CREBZF* | 4039.280 | 0.295 | 0.0007 | 0.0133 |
| *HNRNPH1* | 16470.605 | 0.296 | 0.0004 | 0.0094 |
| *P4HTM* | 2991.430 | 0.297 | 5.56E-05 | 0.0027 |
| *FANCA* | 705.193 | 0.297 | 0.0032 | 0.0358 |
| *CCNO* | 10896.667 | 0.299 | 0.0008 | 0.0144 |
| *PPP1R12C* | 2853.339 | 0.300 | 0.0019 | 0.0265 |
| *SPSB3* | 1715.851 | 0.301 | 0.0006 | 0.0130 |
| *ELMOD3* | 1008.952 | 0.301 | 0.0010 | 0.0174 |
| *WSB1* | 5661.202 | 0.301 | 0.0016 | 0.0239 |
| *E4F1* | 1195.493 | 0.301 | 0.0015 | 0.0227 |
| *ZNF133* | 727.552 | 0.301 | 0.0053 | 0.0480 |
| *HSPE1* | 523.595 | 0.302 | 0.0056 | 0.0494 |
| *MAN2C1* | 3016.566 | 0.302 | 0.0038 | 0.0395 |
| *SAMD10* | 706.819 | 0.302 | 0.0056 | 0.0494 |
| *OSGEP* | 808.568 | 0.303 | 0.0024 | 0.0304 |
| *C19orf66* | 1152.816 | 0.303 | 0.0021 | 0.0275 |
| *RBM5* | 5453.387 | 0.304 | 0.0008 | 0.0154 |
| *PHYKPL* | 1069.157 | 0.305 | 0.0056 | 0.0493 |
| *MBOAT1* | 2555.049 | 0.305 | 6.06E-05 | 0.0029 |
| *SLFN13* | 1688.975 | 0.305 | 0.0001 | 0.0048 |
| *GAS8* | 1826.035 | 0.306 | 0.0002 | 0.0057 |
| *PHF11* | 1001.433 | 0.306 | 0.0018 | 0.0251 |
| *CLK4* | 922.529 | 0.306 | 0.0044 | 0.0427 |
| *ULK3* | 1545.720 | 0.306 | 0.0006 | 0.0117 |
| *INTS3* | 4437.947 | 0.307 | 0.0014 | 0.0210 |
| *YPEL3* | 2201.495 | 0.308 | 0.0007 | 0.0140 |
| *SRRT* | 5880.613 | 0.308 | 0.0001 | 0.0052 |
| *NLRP1* | 896.393 | 0.309 | 0.0048 | 0.0451 |
| *CLSTN3* | 2079.377 | 0.311 | 0.0035 | 0.0372 |
| *INPPL1* | 5187.771 | 0.311 | 0.0016 | 0.0230 |
| *SLC4A11* | 7128.218 | 0.311 | 0.0021 | 0.0276 |
| *INPP5E* | 1242.310 | 0.311 | 0.0008 | 0.0147 |
| *PRPF3* | 1869.643 | 0.312 | 0.0002 | 0.0058 |
| *MRPL55* | 681.846 | 0.312 | 0.0011 | 0.0176 |
| *TMEM175* | 1164.117 | 0.312 | 0.0024 | 0.0299 |
| *ANAPC4* | 1138.867 | 0.313 | 0.0008 | 0.0153 |
| *PLEKHG5* | 2015.993 | 0.313 | 0.0040 | 0.0406 |
| *THAP9-AS1* | 2295.811 | 0.315 | 0.0001 | 0.0049 |
| *MUC4* | 50161.072 | 0.317 | 8.69E-06 | 0.0008 |
| *SFXN4* | 820.347 | 0.317 | 0.0046 | 0.0441 |
| *CHKA* | 2268.722 | 0.317 | 0.0005 | 0.0117 |
| *SPPL2B* | 3337.683 | 0.317 | 0.0011 | 0.0183 |
| *ZNF688* | 673.415 | 0.319 | 0.0010 | 0.0164 |
| *TIA1* | 5394.574 | 0.320 | 0.0008 | 0.0154 |
| *SIRT7* | 1336.621 | 0.321 | 0.0006 | 0.0122 |
| *NXF1* | 4067.905 | 0.321 | 0.0009 | 0.0162 |
| *STK33* | 892.717 | 0.321 | 0.0017 | 0.0247 |
| *SYNE4* | 578.419 | 0.322 | 0.0010 | 0.0166 |
| *PSENEN* | 3364.617 | 0.323 | 0.0010 | 0.0164 |
| *SGSM2* | 7857.202 | 0.323 | 0.0008 | 0.0153 |
| *SRSF5* | 12196.477 | 0.323 | 0.0002 | 0.0057 |
| *ARRDC1* | 3380.433 | 0.324 | 0.0003 | 0.0080 |
| *PKD1* | 3704.934 | 0.324 | 0.0048 | 0.0449 |
| *DPH7* | 1122.990 | 0.324 | 0.0004 | 0.0102 |
| *KAT2A* | 1835.589 | 0.324 | 0.0011 | 0.0183 |
| *MIB2* | 1731.519 | 0.325 | 0.0049 | 0.0455 |
| *MBD6* | 5248.425 | 0.325 | 0.0025 | 0.0308 |
| *ALKBH6* | 555.929 | 0.326 | 0.0034 | 0.0369 |
| *ZNF302* | 1170.662 | 0.326 | 0.0007 | 0.0138 |
| *CCDC64B* | 4412.953 | 0.326 | 0.0006 | 0.0128 |
| *LUC7L3* | 7541.872 | 0.326 | 0.0007 | 0.0137 |
| *DNALI1* | 2795.133 | 0.326 | 0.0004 | 0.0095 |
| *LPPR2* | 956.363 | 0.327 | 0.0012 | 0.0194 |
| *LRRC23* | 2620.462 | 0.328 | 0.0027 | 0.0327 |
| *SRRM2* | 37404.739 | 0.328 | 0.0009 | 0.0157 |
| *RAPGEFL1* | 6529.396 | 0.328 | 0.0016 | 0.0232 |
| *ANO8* | 1415.493 | 0.328 | 0.0041 | 0.0412 |
| *ARRDC2* | 1250.505 | 0.330 | 0.0003 | 0.0088 |
| *MAATS1* | 902.391 | 0.330 | 0.0014 | 0.0210 |
| *MYEF2* | 672.694 | 0.330 | 0.0034 | 0.0370 |
| *QTRT1* | 1205.034 | 0.330 | 0.0018 | 0.0257 |
| *FNBP4* | 3784.214 | 0.330 | 0.0005 | 0.0113 |
| *WDR90* | 2630.759 | 0.331 | 0.0004 | 0.0097 |
| *ARHGAP40* | 1778.953 | 0.331 | 0.0009 | 0.0158 |
| *DHX34* | 992.148 | 0.332 | 0.0008 | 0.0154 |
| *CLK1* | 3855.592 | 0.332 | 0.0014 | 0.0211 |
| *RBM41* | 1759.934 | 0.332 | 0.0007 | 0.0133 |
| *MAPK11* | 550.584 | 0.332 | 0.0041 | 0.0412 |
| *ADM* | 535.721 | 0.332 | 0.0024 | 0.0303 |
| *AP1G2* | 5729.398 | 0.333 | 0.0005 | 0.0108 |
| *FCHSD1* | 820.145 | 0.334 | 0.0023 | 0.0293 |
| *CCDC24* | 1120.790 | 0.335 | 0.0012 | 0.0186 |
| *ZNF300* | 649.394 | 0.335 | 0.0036 | 0.0380 |
| *NAPB* | 811.063 | 0.335 | 0.0047 | 0.0444 |
| *CNKSR1* | 1790.471 | 0.337 | 0.0002 | 0.0058 |
| *SETD4* | 1047.187 | 0.337 | 0.0011 | 0.0183 |
| *PARP6* | 1857.905 | 0.337 | 0.0006 | 0.0130 |
| *ENKD1* | 1011.391 | 0.337 | 7.03E-05 | 0.0032 |
| *ZNF514* | 809.340 | 0.338 | 0.0027 | 0.0321 |
| *ASB16-AS1* | 571.494 | 0.339 | 0.0033 | 0.0365 |
| *KIAA0907* | 2815.260 | 0.339 | 0.0005 | 0.0108 |
| *MST1R* | 3178.767 | 0.340 | 0.0009 | 0.0158 |
| *ANKZF1* | 2627.991 | 0.340 | 3.71E-06 | 0.0005 |
| *PHKG2* | 1158.657 | 0.340 | 0.0001 | 0.0047 |
| *WDR73* | 1467.102 | 0.340 | 0.0001 | 0.0046 |
| *DNAAF3* | 2669.148 | 0.341 | 0.0021 | 0.0275 |
| *NSUN6* | 438.735 | 0.342 | 0.0044 | 0.0425 |
| *MAN2A2* | 1586.604 | 0.342 | 0.0006 | 0.0118 |
| *TRA2A* | 4055.516 | 0.342 | 1.40E-05 | 0.0012 |
| *ZNF160* | 1122.731 | 0.343 | 0.0005 | 0.0106 |
| *RSPH1* | 3601.300 | 0.343 | 0.0021 | 0.0275 |
| *CCDC14* | 3655.853 | 0.343 | 0.0010 | 0.0164 |
| *MUTYH* | 440.396 | 0.343 | 0.0046 | 0.0441 |
| *AKAP8L* | 3796.044 | 0.344 | 0.0005 | 0.0108 |
| *PAN2* | 1865.971 | 0.344 | 0.0013 | 0.0201 |
| *MS4A8* | 730.717 | 0.345 | 0.0015 | 0.0226 |
| *SNAPC4* | 1429.725 | 0.345 | 0.0011 | 0.0175 |
| *C7orf63* | 773.973 | 0.345 | 0.0011 | 0.0176 |
| *FBF1* | 708.626 | 0.346 | 0.0040 | 0.0404 |
| *ANKRD10* | 3091.267 | 0.346 | 0.0006 | 0.0127 |
| *CDK20* | 588.870 | 0.348 | 0.0037 | 0.0388 |
| *LMBR1L* | 929.805 | 0.348 | 0.0047 | 0.0444 |
| *SFI1* | 1012.165 | 0.348 | 0.0001 | 0.0053 |
| *KIAA1407* | 970.279 | 0.349 | 0.0003 | 0.0088 |
| *THOC1* | 1061.946 | 0.350 | 6.67E-05 | 0.0031 |
| *ANKRD36C* | 738.987 | 0.350 | 0.0006 | 0.0122 |
| *ARGLU1* | 9799.656 | 0.350 | 0.0005 | 0.0113 |
| *UBA7* | 2745.962 | 0.350 | 9.68E-05 | 0.0040 |
| *ENGASE* | 1580.495 | 0.350 | 0.0018 | 0.0252 |
| *BCS1L* | 1055.414 | 0.351 | 0.0003 | 0.0082 |
| *KCTD13* | 550.246 | 0.351 | 0.0018 | 0.0251 |
| *PYROXD2* | 1066.286 | 0.353 | 0.0029 | 0.0336 |
| *CAPS2* | 562.342 | 0.354 | 0.0013 | 0.0203 |
| *NPNT* | 1271.531 | 0.354 | 4.37E-05 | 0.0023 |
| *STX16* | 4447.095 | 0.355 | 4.48E-05 | 0.0024 |
| *TCIRG1* | 5751.388 | 0.355 | 0.0002 | 0.0056 |
| *PSMC3IP* | 400.996 | 0.356 | 0.0019 | 0.0259 |
| *METTL17* | 1939.555 | 0.356 | 0.0001 | 0.0047 |
| *C9orf116* | 1114.095 | 0.356 | 0.0017 | 0.0239 |
| *CCDC74A* | 2246.539 | 0.358 | 0.0004 | 0.0096 |
| *SYNGAP1* | 1481.126 | 0.358 | 0.0011 | 0.0184 |
| *TNRC6C-AS1* | 691.255 | 0.359 | 0.0040 | 0.0404 |
| *EPS8L1* | 8815.059 | 0.359 | 4.20E-05 | 0.0023 |
| *ENDOV* | 348.422 | 0.359 | 0.0029 | 0.0337 |
| *WDR16* | 701.399 | 0.360 | 0.0008 | 0.0148 |
| *XIST* | 11748.744 | 0.361 | 0.0018 | 0.0250 |
| *ZMYND15* | 403.371 | 0.361 | 0.0021 | 0.0278 |
| *LRRC56* | 841.786 | 0.362 | 7.94E-05 | 0.0034 |
| *SNTN* | 1553.117 | 0.362 | 0.0032 | 0.0357 |
| *FAAH* | 1373.038 | 0.362 | 0.0002 | 0.0064 |
| *USP43* | 900.873 | 0.362 | 0.0048 | 0.0451 |
| *WNT4* | 3001.774 | 0.363 | 2.75E-05 | 0.0018 |
| *PRPF39* | 1479.373 | 0.364 | 0.0002 | 0.0062 |
| *PNN* | 6681.950 | 0.364 | 3.47E-05 | 0.0020 |
| *TTC25* | 853.581 | 0.365 | 0.0025 | 0.0309 |
| *SNHG1* | 1570.789 | 0.366 | 0.0005 | 0.0108 |
| *MORN1* | 328.834 | 0.367 | 0.0055 | 0.0491 |
| *PIDD* | 1185.582 | 0.367 | 0.0025 | 0.0308 |
| *IPW* | 691.819 | 0.368 | 0.0047 | 0.0442 |
| *OSBPL7* | 1239.790 | 0.368 | 0.0004 | 0.0099 |
| *MAP6* | 797.380 | 0.369 | 0.0041 | 0.0409 |
| *RASSF7* | 2407.252 | 0.369 | 0.0004 | 0.0095 |
| *ABO* | 1203.729 | 0.369 | 0.0033 | 0.0361 |
| *MTRF1* | 621.015 | 0.369 | 0.0005 | 0.0108 |
| *HPRT1* | 680.283 | 0.369 | 6.86E-05 | 0.0031 |
| *PLXNB3* | 1859.010 | 0.371 | 0.0003 | 0.0087 |
| *TRIM66* | 972.142 | 0.372 | 0.0041 | 0.0412 |
| *ABCC5* | 4159.816 | 0.372 | 0.0014 | 0.0210 |
| *ZNF862* | 1203.475 | 0.373 | 0.0008 | 0.0144 |
| *ENOSF1* | 2313.872 | 0.373 | 1.35E-05 | 0.0012 |
| *ABCA5* | 2038.285 | 0.373 | 0.0003 | 0.0080 |
| *HEXDC* | 1327.467 | 0.374 | 0.0033 | 0.0361 |
| *CENPT* | 2032.157 | 0.374 | 0.0002 | 0.0064 |
| *FANK1* | 1018.891 | 0.375 | 0.0002 | 0.0065 |
| *IGFBP3* | 38742.302 | 0.375 | 0.0001 | 0.0049 |
| *PASK* | 1275.584 | 0.375 | 6.14E-05 | 0.0029 |
| *CCDC159* | 474.832 | 0.376 | 0.0025 | 0.0310 |
| *STK36* | 1827.743 | 0.376 | 3.15E-05 | 0.0019 |
| *TUBGCP6* | 2896.053 | 0.377 | 9.62E-05 | 0.0040 |
| *LOC101928817* | 837.720 | 0.377 | 0.0022 | 0.0281 |
| *TP53I13* | 1583.250 | 0.378 | 0.0002 | 0.0057 |
| *TSPYL2* | 993.234 | 0.378 | 0.0003 | 0.0085 |
| *SEPT7P2* | 306.738 | 0.378 | 0.0039 | 0.0401 |
| *ZNF337* | 838.201 | 0.379 | 0.0007 | 0.0131 |
| *MAPK10* | 1243.844 | 0.379 | 4.82E-06 | 0.0006 |
| *C9orf24* | 1298.442 | 0.379 | 5.05E-05 | 0.0025 |
| *EFCAB4A* | 5084.645 | 0.379 | 0.0005 | 0.0107 |
| *C9orf142* | 578.718 | 0.379 | 0.0021 | 0.0279 |
| *RBM6* | 4419.992 | 0.380 | 0.0001 | 0.0047 |
| *PNISR* | 7378.550 | 0.381 | 7.61E-05 | 0.0033 |
| *CCDC142* | 510.373 | 0.381 | 0.0020 | 0.0271 |
| *LRRC46* | 2453.143 | 0.381 | 0.0009 | 0.0155 |
| *XAF1* | 1485.597 | 0.382 | 0.0007 | 0.0142 |
| *MT1E* | 441.204 | 0.382 | 0.0026 | 0.0316 |
| *CCNL1* | 4819.061 | 0.382 | 0.0004 | 0.0103 |
| *ADAT2* | 537.480 | 0.383 | 0.0024 | 0.0299 |
| *GPSM1* | 451.203 | 0.383 | 0.0032 | 0.0355 |
| *DMTF1* | 1917.157 | 0.384 | 0.0007 | 0.0136 |
| *LOC100506990* | 286.703 | 0.384 | 0.0040 | 0.0404 |
| *FHOD1* | 679.377 | 0.384 | 0.0010 | 0.0166 |
| *LRRC48* | 1097.089 | 0.385 | 0.0002 | 0.0067 |
| *GIGYF1* | 3990.523 | 0.385 | 0.0009 | 0.0163 |
| *DLG4* | 866.601 | 0.385 | 0.0013 | 0.0199 |
| *DDX26B* | 578.908 | 0.387 | 0.0024 | 0.0301 |
| *TRMT1* | 1003.018 | 0.388 | 0.0004 | 0.0091 |
| *SDHAP1* | 461.410 | 0.388 | 0.0016 | 0.0233 |
| *STAG3L2* | 237.534 | 0.388 | 0.0043 | 0.0419 |
| *RP11-395P17.3* | 876.139 | 0.389 | 0.0018 | 0.0257 |
| *ZNF251* | 716.865 | 0.389 | 0.0002 | 0.0067 |
| *FMO6P* | 1239.298 | 0.389 | 1.52E-05 | 0.0012 |
| *SH2B1* | 3696.178 | 0.390 | 0.0002 | 0.0064 |
| *GAS2L2* | 1083.931 | 0.392 | 0.0018 | 0.0253 |
| *NBPF15* | 647.620 | 0.393 | 0.0021 | 0.0278 |
| *CLK2* | 2327.654 | 0.393 | 0.0004 | 0.0093 |
| *WDR86-AS1* | 737.102 | 0.394 | 0.0020 | 0.0269 |
| *FAM183A* | 708.255 | 0.395 | 0.0007 | 0.0137 |
| *IRF3* | 3616.829 | 0.395 | 1.82E-06 | 0.0003 |
| *ESPN* | 2263.037 | 0.395 | 6.19E-05 | 0.0029 |
| *PLXNA3* | 1989.609 | 0.395 | 0.0003 | 0.0087 |
| *ZNF266* | 1614.987 | 0.395 | 0.0004 | 0.0097 |
| *CDK5RAP3* | 5500.465 | 0.396 | 4.23E-05 | 0.0023 |
| *ZNF354B* | 374.103 | 0.396 | 0.0025 | 0.0306 |
| *LOC80154* | 3298.030 | 0.397 | 0.0003 | 0.0078 |
| *C20orf96* | 1162.359 | 0.397 | 4.82E-06 | 0.0006 |
| *THBS3* | 845.972 | 0.398 | 0.0031 | 0.0352 |
| *SLC25A29* | 5945.887 | 0.398 | 6.21E-06 | 0.0007 |
| *THUMPD3-AS1* | 683.442 | 0.398 | 2.44E-05 | 0.0016 |
| *HES4* | 3237.240 | 0.400 | 7.93E-06 | 0.0008 |
| *PLA2G6* | 721.265 | 0.401 | 0.0046 | 0.0442 |
| *UVSSA* | 1123.004 | 0.401 | 0.0010 | 0.0164 |
| *ICA1L* | 412.502 | 0.404 | 0.0007 | 0.0131 |
| *LOC728392* | 288.634 | 0.404 | 0.0022 | 0.0288 |
| *ANKRD37* | 317.691 | 0.405 | 0.0025 | 0.0308 |
| *CCDC74B* | 756.792 | 0.406 | 0.0003 | 0.0083 |
| *TNK2* | 1828.400 | 0.407 | 0.0002 | 0.0061 |
| *TAF1C* | 2165.446 | 0.407 | 0.0003 | 0.0086 |
| *CCDC146* | 5497.474 | 0.407 | 2.39E-08 | 1.28E-05 |
| *ZNF276* | 1450.286 | 0.407 | 0.0004 | 0.0099 |
| *CCDC135* | 968.870 | 0.407 | 0.0006 | 0.0130 |
| *DBF4B* | 400.767 | 0.408 | 0.0003 | 0.0086 |
| *FLJ31306* | 1925.237 | 0.409 | 0.0004 | 0.0096 |
| *TROAP* | 640.040 | 0.410 | 0.0018 | 0.0257 |
| *TAZ* | 982.675 | 0.410 | 0.0005 | 0.0108 |
| *LRRIQ1* | 3026.368 | 0.412 | 0.0004 | 0.0093 |
| *LOC150776* | 695.922 | 0.412 | 0.0028 | 0.0332 |
| *AMN* | 522.432 | 0.413 | 0.0011 | 0.0176 |
| *ADPRHL2* | 1449.616 | 0.413 | 0.0005 | 0.0107 |
| *CNTN3* | 404.590 | 0.413 | 0.0006 | 0.0125 |
| *CDK11A* | 454.091 | 0.413 | 0.0017 | 0.0248 |
| *MORN2* | 1116.508 | 0.414 | 2.67E-05 | 0.0017 |
| *TTLL10* | 890.540 | 0.414 | 2.72E-05 | 0.0018 |
| *C17orf72* | 1395.099 | 0.415 | 3.58E-05 | 0.0021 |
| *LOC102723726* | 855.976 | 0.416 | 0.0007 | 0.0136 |
| *UCKL1* | 1462.540 | 0.417 | 4.97E-05 | 0.0025 |
| *ZNF793* | 476.658 | 0.417 | 9.36E-05 | 0.0039 |
| *LOC100506603* | 250.009 | 0.418 | 0.0039 | 0.0400 |
| *PCSK4* | 473.803 | 0.418 | 0.0018 | 0.0251 |
| *COL27A1* | 1107.272 | 0.419 | 0.0028 | 0.0331 |
| *PLXNB1* | 14992.980 | 0.420 | 6.49E-05 | 0.0030 |
| *TPM2* | 3606.506 | 0.420 | 1.17E-05 | 0.0010 |
| *U2AF1L4* | 350.067 | 0.420 | 0.0044 | 0.0427 |
| *COLCA1* | 2475.026 | 0.420 | 3.06E-08 | 1.43E-05 |
| *SLC26A6* | 1040.666 | 0.421 | 0.0001 | 0.0042 |
| *FOXN4* | 1705.998 | 0.421 | 0.0004 | 0.0100 |
| *NOXA1* | 1235.260 | 0.422 | 0.0011 | 0.0180 |
| *TNFRSF14* | 4755.042 | 0.422 | 4.69E-05 | 0.0024 |
| *HSBP1L1* | 1328.501 | 0.423 | 1.14E-06 | 0.0002 |
| *MEGF6* | 1319.568 | 0.423 | 0.0020 | 0.0272 |
| *RNPC3* | 718.639 | 0.424 | 3.09E-05 | 0.0019 |
| *WDR96* | 2506.260 | 0.424 | 7.58E-05 | 0.0033 |
| *MIR600HG* | 526.440 | 0.424 | 0.0013 | 0.0203 |
| *HERC2P2* | 1235.987 | 0.425 | 0.0009 | 0.0156 |
| *PLEKHM1P* | 593.056 | 0.426 | 0.0014 | 0.0209 |
| *MOK* | 1175.999 | 0.426 | 9.49E-05 | 0.0039 |
| *GLI4* | 562.023 | 0.426 | 0.0010 | 0.0174 |
| *EFCAB12* | 747.249 | 0.427 | 0.0016 | 0.0228 |
| *RABL2A* | 1098.214 | 0.428 | 6.70E-06 | 0.0007 |
| *CCDC11* | 560.241 | 0.428 | 0.0003 | 0.0087 |
| *CDH24* | 1038.892 | 0.429 | 0.0003 | 0.0076 |
| *PRICKLE4* | 2018.106 | 0.429 | 0.0002 | 0.0054 |
| *MORN5* | 431.893 | 0.429 | 0.0008 | 0.0153 |
| *CROCCP2* | 1090.511 | 0.430 | 0.0001 | 0.0049 |
| *C1QTNF6* | 288.556 | 0.430 | 0.0032 | 0.0355 |
| *RRAD* | 2101.798 | 0.433 | 0.0003 | 0.0086 |
| *HIST1H1C* | 1626.912 | 0.434 | 6.60E-06 | 0.0007 |
| *TMEM234* | 756.190 | 0.435 | 0.0010 | 0.0166 |
| *LUC7L* | 2850.625 | 0.435 | 1.41E-05 | 0.0012 |
| *DECR2* | 425.689 | 0.435 | 0.0008 | 0.0149 |
| *FAM179A* | 552.803 | 0.436 | 0.0002 | 0.0071 |
| *TTC40* | 1532.989 | 0.436 | 4.13E-05 | 0.0022 |
| *CLASRP* | 1827.568 | 0.437 | 0.0001 | 0.0047 |
| *LTB4R* | 1954.710 | 0.437 | 0.0002 | 0.0057 |
| *CCDC40* | 1362.714 | 0.438 | 7.92E-07 | 0.0002 |
| *C1orf159* | 1041.571 | 0.439 | 0.0016 | 0.0231 |
| *MYL5* | 385.493 | 0.439 | 0.0025 | 0.0309 |
| *TUBG2* | 444.287 | 0.439 | 0.0030 | 0.0343 |
| *C21orf58* | 1633.781 | 0.440 | 2.00E-07 | 5.94E-05 |
| *CCT6P1* | 228.337 | 0.440 | 0.0051 | 0.0470 |
| *ADAM1A* | 241.456 | 0.441 | 0.0033 | 0.0361 |
| *CATSPERB* | 423.148 | 0.441 | 0.0031 | 0.0352 |
| *NKTR* | 5758.206 | 0.443 | 2.84E-05 | 0.0018 |
| *SPATA6L* | 662.672 | 0.443 | 1.09E-05 | 0.0010 |
| *TMEM184A* | 3373.268 | 0.444 | 0.0001 | 0.0043 |
| *RP11-242D8.1* | 211.135 | 0.444 | 0.0035 | 0.0374 |
| *FAM92B* | 1385.769 | 0.445 | 2.83E-05 | 0.0018 |
| *TEKT2* | 1746.264 | 0.445 | 1.77E-05 | 0.0013 |
| *CAPRIN2* | 775.820 | 0.446 | 7.87E-06 | 0.0008 |
| *MXD3* | 502.990 | 0.446 | 0.0004 | 0.0097 |
| *CD164L2* | 404.030 | 0.446 | 0.0005 | 0.0106 |
| *FAM166B* | 758.055 | 0.447 | 0.0002 | 0.0064 |
| *C5orf45* | 569.897 | 0.447 | 0.0002 | 0.0057 |
| *HCN3* | 383.934 | 0.447 | 0.0036 | 0.0380 |
| *CCDC57* | 1664.004 | 0.449 | 1.95E-05 | 0.0014 |
| *LOC286437* | 260.034 | 0.449 | 0.0052 | 0.0474 |
| *CDK10* | 2465.168 | 0.449 | 9.68E-06 | 0.0009 |
| *TRPV1* | 399.250 | 0.450 | 0.0028 | 0.0333 |
| *KIAA1751* | 1147.541 | 0.450 | 0.0002 | 0.0055 |
| *SNHG3* | 708.014 | 0.451 | 1.07E-05 | 0.0010 |
| *SNED1* | 280.221 | 0.451 | 0.0022 | 0.0283 |
| *PPP1R32* | 565.845 | 0.451 | 9.89E-05 | 0.0041 |
| *KIAA0895L* | 1328.739 | 0.452 | 0.0003 | 0.0084 |
| *KLHL17* | 624.573 | 0.452 | 0.0020 | 0.0273 |
| *D2HGDH* | 2157.028 | 0.453 | 0.0001 | 0.0046 |
| *ODF3B* | 1674.782 | 0.453 | 2.43E-06 | 0.0004 |
| *CHTF18* | 1106.682 | 0.454 | 0.0001 | 0.0048 |
| *CCDC42B* | 1679.412 | 0.456 | 2.62E-06 | 0.0004 |
| *PPIEL* | 434.360 | 0.456 | 0.0037 | 0.0389 |
| *C1RL-AS1* | 332.981 | 0.457 | 0.0021 | 0.0276 |
| *MICALL2* | 5005.373 | 0.457 | 1.65E-05 | 0.0013 |
| *LPIN3* | 1500.670 | 0.457 | 0.0002 | 0.0057 |
| *PRKXP1* | 271.709 | 0.457 | 0.0019 | 0.0259 |
| *INO80E* | 3040.884 | 0.458 | 4.78E-06 | 0.0006 |
| *CYP2B7P* | 15481.573 | 0.458 | 7.39E-07 | 0.0002 |
| *IDUA* | 1434.591 | 0.459 | 2.28E-05 | 0.0016 |
| *NTF4* | 298.834 | 0.459 | 0.0007 | 0.0136 |
| *UBXN11* | 2797.179 | 0.460 | 1.76E-07 | 5.46E-05 |
| *MZF1* | 1570.088 | 0.460 | 3.07E-06 | 0.0005 |
| *CROCC* | 3046.313 | 0.460 | 3.35E-05 | 0.0020 |
| *APBB3* | 904.504 | 0.462 | 0.0001 | 0.0046 |
| *C4orf22* | 228.639 | 0.462 | 0.0047 | 0.0446 |
| *ZNF23* | 748.196 | 0.464 | 0.0002 | 0.0060 |
| *WDR52* | 2063.139 | 0.465 | 1.23E-07 | 4.23E-05 |
| *TUBA4B* | 942.084 | 0.467 | 0.0008 | 0.0147 |
| *ZNF10* | 626.964 | 0.468 | 0.0007 | 0.0132 |
| *GIPR* | 296.419 | 0.468 | 0.0041 | 0.0407 |
| *ZNF37BP* | 763.028 | 0.469 | 0.0002 | 0.0062 |
| *SLC25A35* | 279.382 | 0.470 | 0.0041 | 0.0407 |
| *RP11-696N14.1* | 650.786 | 0.472 | 0.0002 | 0.0058 |
| *CNGA4* | 230.792 | 0.472 | 0.0019 | 0.0258 |
| *RP11-503N18.1* | 466.991 | 0.472 | 0.0002 | 0.0057 |
| *ADAMTSL4* | 235.878 | 0.472 | 0.0028 | 0.0333 |
| *RHPN1* | 2051.711 | 0.472 | 2.05E-05 | 0.0015 |
| *SNHG10* | 181.637 | 0.475 | 0.0019 | 0.0265 |
| *CCDC157* | 829.417 | 0.475 | 3.79E-05 | 0.0021 |
| *SH3BP5-AS1* | 512.424 | 0.475 | 0.0028 | 0.0330 |
| *CLHC1* | 467.968 | 0.475 | 0.0006 | 0.0117 |
| *CCDC108* | 840.190 | 0.477 | 1.68E-05 | 0.0013 |
| *CCDC151* | 695.571 | 0.477 | 4.67E-05 | 0.0024 |
| *LAMA4* | 206.021 | 0.478 | 0.0056 | 0.0495 |
| *NLGN2* | 733.889 | 0.479 | 0.0004 | 0.0093 |
| *LRRC43* | 279.438 | 0.479 | 0.0004 | 0.0104 |
| *ANKRD18B* | 457.481 | 0.480 | 0.0010 | 0.0170 |
| *LOC100288778* | 482.141 | 0.481 | 0.0014 | 0.0214 |
| *ACCS* | 636.779 | 0.481 | 0.0003 | 0.0086 |
| *ATAT1* | 975.459 | 0.481 | 1.62E-05 | 0.0013 |
| *LINC00969* | 144.268 | 0.482 | 0.0047 | 0.0442 |
| *ATAD3B* | 813.730 | 0.483 | 0.0003 | 0.0076 |
| *ANKDD1B* | 159.514 | 0.484 | 0.0033 | 0.0360 |
| *FAM193B* | 2295.898 | 0.484 | 6.33E-05 | 0.0030 |
| *DENND6B* | 992.494 | 0.485 | 1.86E-08 | 1.04E-05 |
| *MDM4* | 1848.130 | 0.486 | 0.0001 | 0.0047 |
| *DNAI2* | 929.814 | 0.487 | 6.40E-07 | 0.0001 |
| *C2orf62* | 425.629 | 0.488 | 0.0004 | 0.0096 |
| *ENTHD2* | 1330.549 | 0.488 | 0.0003 | 0.0081 |
| *TTC21A* | 1119.298 | 0.489 | 2.05E-05 | 0.0015 |
| *ZNF547* | 157.894 | 0.490 | 0.0032 | 0.0357 |
| *ZNF117* | 1305.132 | 0.491 | 1.17E-05 | 0.0010 |
| *C1orf63* | 4879.435 | 0.491 | 2.59E-06 | 0.0004 |
| *CNTNAP1* | 175.030 | 0.491 | 0.0019 | 0.0258 |
| *ADHFE1* | 544.407 | 0.492 | 0.0003 | 0.0083 |
| *ZNF767* | 723.709 | 0.492 | 2.82E-05 | 0.0018 |
| *LRGUK* | 253.694 | 0.492 | 0.0006 | 0.0117 |
| *RASSF4* | 1068.418 | 0.493 | 6.32E-06 | 0.0007 |
| *MDH1B* | 448.353 | 0.493 | 0.0002 | 0.0069 |
| *ANKRD20A5P* | 298.584 | 0.494 | 0.0010 | 0.0169 |
| *SCARF1* | 197.666 | 0.495 | 0.0046 | 0.0440 |
| *HNRNPU-AS1* | 1340.853 | 0.495 | 2.64E-05 | 0.0017 |
| *SPAG17* | 1417.685 | 0.495 | 2.79E-05 | 0.0018 |
| *AMT* | 510.541 | 0.496 | 0.0003 | 0.0082 |
| *ZNF692* | 1748.274 | 0.496 | 7.73E-05 | 0.0034 |
| *PCOLCE* | 219.736 | 0.496 | 0.0014 | 0.0209 |
| *ANPEP* | 2298.169 | 0.498 | 6.85E-07 | 0.0002 |
| *CCDC153* | 365.581 | 0.498 | 7.97E-05 | 0.0034 |
| *PABPN1* | 573.510 | 0.498 | 0.0002 | 0.0054 |
| *LINC01089* | 746.101 | 0.500 | 6.72E-06 | 0.0007 |
| *TP53AIP1* | 399.631 | 0.501 | 4.49E-05 | 0.0024 |
| *BCO2* | 288.890 | 0.501 | 0.0001 | 0.0047 |
| *MROH9* | 325.382 | 0.502 | 0.0043 | 0.0423 |
| *LOC642846* | 301.806 | 0.503 | 0.0012 | 0.0186 |
| *LOC202181* | 182.077 | 0.503 | 0.0032 | 0.0355 |
| *ANKS3* | 604.250 | 0.503 | 3.36E-06 | 0.0005 |
| *ZNF83* | 1390.346 | 0.503 | 4.79E-07 | 1.16E-04 |
| *DPY19L2* | 404.160 | 0.504 | 0.0024 | 0.0303 |
| *CRIPAK* | 658.685 | 0.504 | 0.0003 | 0.0090 |
| *DFNB31* | 858.179 | 0.506 | 1.24E-07 | 4.23E-05 |
| *EME2* | 325.448 | 0.508 | 0.0002 | 0.0061 |
| *ANKRD36* | 141.732 | 0.508 | 0.0038 | 0.0391 |
| *ADAMTS13* | 216.369 | 0.512 | 0.0044 | 0.0427 |
| *NOL3* | 1055.716 | 0.513 | 7.35E-06 | 0.0008 |
| *ECHDC2* | 2116.578 | 0.513 | 3.63E-06 | 0.0005 |
| *LOC401320* | 288.425 | 0.515 | 0.0015 | 0.0221 |
| *EFHC1* | 3039.980 | 0.515 | 2.42E-07 | 6.51E-05 |
| *NME9* | 371.142 | 0.516 | 0.0002 | 0.0058 |
| *LOC155060* | 502.381 | 0.516 | 0.0007 | 0.0132 |
| *SH3D21* | 1081.307 | 0.518 | 8.26E-06 | 0.0008 |
| *HDAC10* | 1532.393 | 0.518 | 1.00E-05 | 0.0009 |
| *NEIL1* | 1469.508 | 0.519 | 2.09E-05 | 0.0015 |
| *RP11-384K6.6* | 445.641 | 0.520 | 0.0001 | 0.0043 |
| *FAM20A* | 555.220 | 0.520 | 9.41E-05 | 0.0039 |
| *C1orf194* | 635.471 | 0.521 | 1.69E-05 | 0.0013 |
| *GDPD3* | 174.440 | 0.521 | 0.0024 | 0.0301 |
| *SYT5* | 934.741 | 0.521 | 0.0002 | 0.0057 |
| *DNAH6* | 1060.652 | 0.522 | 2.78E-05 | 0.0018 |
| *ATHL1* | 2450.282 | 0.526 | 7.02E-06 | 0.0007 |
| *PPP1R26-AS1* | 152.337 | 0.526 | 0.0030 | 0.0344 |
| *LRP5L* | 186.270 | 0.526 | 0.0052 | 0.0473 |
| *PNPLA7* | 525.067 | 0.528 | 5.09E-05 | 0.0026 |
| *PABPC1L* | 1231.246 | 0.529 | 3.28E-05 | 0.0019 |
| *PI4KAP2* | 155.368 | 0.529 | 0.0025 | 0.0305 |
| *DYNLRB2* | 252.543 | 0.530 | 0.0005 | 0.0109 |
| *LOC100507577* | 356.846 | 0.530 | 4.35E-05 | 0.0023 |
| *CXorf30* | 494.786 | 0.532 | 0.0006 | 0.0130 |
| *DERL3* | 505.153 | 0.532 | 1.95E-05 | 0.0014 |
| *ZNF334* | 431.517 | 0.533 | 2.07E-06 | 0.0003 |
| *WDR27* | 1247.359 | 0.534 | 1.17E-05 | 0.0010 |
| *ATG9B* | 271.547 | 0.534 | 0.0003 | 0.0076 |
| *DNAI1* | 1320.761 | 0.534 | 6.04E-06 | 0.0007 |
| *NSUN5P2* | 481.579 | 0.535 | 0.0007 | 0.0133 |
| *NRBP2* | 1917.579 | 0.535 | 1.64E-05 | 0.0013 |
| *SNHG12* | 500.220 | 0.537 | 3.84E-05 | 0.0022 |
| *ITGB2-AS1* | 210.770 | 0.538 | 0.0042 | 0.0414 |
| *TRIM17* | 143.401 | 0.539 | 0.0021 | 0.0280 |
| *RNF207* | 3155.566 | 0.540 | 5.27E-06 | 0.0007 |
| *PDXDC2P* | 1337.281 | 0.540 | 3.73E-06 | 0.0005 |
| *TTC18* | 992.458 | 0.540 | 1.12E-06 | 0.0002 |
| *GSTM2* | 300.587 | 0.541 | 0.0008 | 0.0152 |
| *TTLL3* | 207.546 | 0.542 | 0.0029 | 0.0336 |
| *DFNB59* | 253.638 | 0.543 | 0.0007 | 0.0133 |
| *SCML1* | 444.085 | 0.543 | 1.56E-06 | 0.0003 |
| *CPA4* | 453.344 | 0.544 | 5.02E-05 | 0.0025 |
| *ANKRD23* | 244.765 | 0.545 | 0.0004 | 0.0102 |
| *UPK3B* | 616.395 | 0.547 | 1.34E-05 | 0.0012 |
| *MSANTD2* | 791.355 | 0.548 | 5.89E-06 | 0.0007 |
| *TMEM198* | 185.057 | 0.549 | 0.0030 | 0.0344 |
| *ZMAT1* | 166.414 | 0.549 | 0.0008 | 0.0151 |
| *LOC100233156* | 163.058 | 0.551 | 0.0009 | 0.0162 |
| *NPHP3* | 287.856 | 0.552 | 0.0003 | 0.0077 |
| *RP5-1180C10.2* | 320.206 | 0.552 | 0.0009 | 0.0156 |
| *DDX12P* | 214.940 | 0.553 | 0.0022 | 0.0283 |
| *C5orf66* | 143.534 | 0.554 | 0.0035 | 0.0375 |
| *ERVK13-1* | 482.018 | 0.555 | 3.22E-05 | 0.0019 |
| *GUSBP11* | 608.870 | 0.555 | 3.27E-05 | 0.0019 |
| *WASH3P* | 300.307 | 0.556 | 0.0010 | 0.0166 |
| *CCDC114* | 1641.250 | 0.556 | 8.50E-06 | 0.0008 |
| *TSNAXIP1* | 840.837 | 0.557 | 1.59E-07 | 5.05E-05 |
| *GSDMB* | 952.441 | 0.557 | 5.00E-05 | 0.0025 |
| *IL18BP* | 175.693 | 0.559 | 0.0030 | 0.0344 |
| *GOLGA6L9* | 990.562 | 0.559 | 4.04E-05 | 0.0022 |
| *ZNF577* | 325.757 | 0.559 | 6.65E-05 | 0.0031 |
| *ANO9* | 2065.416 | 0.559 | 2.28E-05 | 0.0016 |
| *L3MBTL1* | 283.225 | 0.560 | 0.0003 | 0.0084 |
| *WASH1* | 193.913 | 0.560 | 0.0025 | 0.0306 |
| *LY6G5B* | 339.225 | 0.561 | 0.0025 | 0.0307 |
| *TTC16* | 198.566 | 0.563 | 0.0005 | 0.0106 |
| *LOC389834* | 229.238 | 0.563 | 0.0002 | 0.0072 |
| *RAD9A* | 1319.840 | 0.563 | 1.08E-05 | 0.0010 |
| *ZNF682* | 234.544 | 0.564 | 0.0001 | 0.0044 |
| *ZNF404* | 126.682 | 0.565 | 0.0031 | 0.0349 |
| *CYP4F12* | 615.784 | 0.565 | 0.0001 | 0.0043 |
| *QRICH2* | 248.980 | 0.565 | 0.0010 | 0.0164 |
| *CCDC84* | 440.495 | 0.567 | 0.0002 | 0.0066 |
| *CLDN15* | 239.628 | 0.569 | 0.0001 | 0.0047 |
| *IZUMO4* | 164.035 | 0.569 | 0.0051 | 0.0467 |
| *C16orf93* | 885.369 | 0.571 | 1.38E-07 | 4.61E-05 |
| *FAM221B* | 202.807 | 0.572 | 0.0005 | 0.0116 |
| *RP11-752G15.6* | 104.483 | 0.572 | 0.0043 | 0.0422 |
| *DNAJB13* | 262.083 | 0.572 | 0.0002 | 0.0057 |
| *C22orf15* | 1026.420 | 0.573 | 2.88E-08 | 1.43E-05 |
| *AIFM3* | 180.529 | 0.573 | 0.0025 | 0.0306 |
| *HAGHL* | 622.327 | 0.573 | 6.90E-08 | 2.68E-05 |
| *LOC284454* | 319.808 | 0.574 | 7.55E-06 | 0.0008 |
| *GABRE* | 4332.953 | 0.574 | 2.25E-07 | 6.42E-05 |
| *LRRC71* | 788.879 | 0.574 | 1.99E-05 | 0.0014 |
| *STAG3L5P-PVRIG2P-PILRB* | 595.817 | 0.577 | 3.95E-05 | 0.0022 |
| *CAPN3* | 776.757 | 0.578 | 5.96E-05 | 0.0028 |
| *DCDC5* | 282.705 | 0.578 | 0.0004 | 0.0104 |
| *WDR93* | 226.899 | 0.579 | 0.0006 | 0.0118 |
| *SNORD80* | 94.288 | 0.581 | 0.0046 | 0.0436 |
| *VAMP1* | 323.229 | 0.581 | 8.33E-05 | 0.0036 |
| *MST1P2* | 234.593 | 0.582 | 0.0021 | 0.0276 |
| *NSUN5P1* | 550.728 | 0.583 | 1.49E-05 | 0.0012 |
| *TMEM86B* | 173.280 | 0.585 | 0.0008 | 0.0147 |
| *CELF6* | 136.047 | 0.586 | 0.0040 | 0.0403 |
| *C1QTNF3* | 93.547 | 0.586 | 0.0041 | 0.0407 |
| *RP11-611D20.2* | 158.995 | 0.587 | 0.0049 | 0.0454 |
| *NUMBL* | 607.940 | 0.589 | 1.20E-05 | 0.0011 |
| *DICER1-AS1* | 137.654 | 0.590 | 0.0018 | 0.0257 |
| *ZFHX2* | 400.769 | 0.593 | 8.69E-06 | 0.0008 |
| *LINC00685* | 234.979 | 0.594 | 0.0037 | 0.0388 |
| *DNAH12* | 2751.674 | 0.595 | 7.87E-07 | 0.0002 |
| *GRIN3B* | 294.863 | 0.595 | 3.66E-05 | 0.0021 |
| *PI4KAP1* | 282.328 | 0.596 | 3.98E-05 | 0.0022 |
| *TTLL9* | 466.620 | 0.597 | 0.0002 | 0.0060 |
| *KCNH3* | 325.047 | 0.598 | 0.0003 | 0.0086 |
| *ALS2CL* | 5674.315 | 0.598 | 0.0050 | 0.0463 |
| *LOC101930275* | 283.553 | 0.599 | 1.59E-05 | 0.0013 |
| *TPT1-AS1* | 300.670 | 0.600 | 0.0001 | 0.0047 |
| *ZNF789* | 243.928 | 0.601 | 2.80E-05 | 0.0018 |
| *IL11RA* | 316.714 | 0.603 | 5.57E-05 | 0.0027 |
| *AGAP4* | 265.576 | 0.603 | 0.0004 | 0.0095 |
| *LOC102724814* | 348.923 | 0.605 | 0.0002 | 0.0068 |
| *PRR22* | 129.203 | 0.605 | 0.0023 | 0.0291 |
| *C11orf35* | 293.777 | 0.607 | 2.68E-05 | 0.0017 |
| *C4orf47* | 409.961 | 0.610 | 1.38E-05 | 0.0012 |
| *MST1* | 832.895 | 0.611 | 1.54E-05 | 0.0012 |
| *LOC100133182* | 230.525 | 0.612 | 0.0018 | 0.0250 |
| *CES4A* | 486.539 | 0.612 | 1.31E-06 | 0.0002 |
| *LOC441124* | 181.778 | 0.612 | 0.0023 | 0.0295 |
| *UNC5CL* | 126.035 | 0.613 | 0.0041 | 0.0408 |
| *HHLA2* | 193.939 | 0.613 | 0.0039 | 0.0396 |
| *LOC115110* | 425.137 | 0.613 | 5.95E-06 | 0.0007 |
| *MIR210HG* | 230.441 | 0.614 | 0.0007 | 0.0137 |
| *LINC00174* | 395.609 | 0.615 | 0.0002 | 0.0058 |
| *H1FX-AS1* | 192.383 | 0.619 | 0.0004 | 0.0093 |
| *CSAD* | 1257.355 | 0.620 | 1.96E-06 | 0.0003 |
| *AGBL2* | 863.677 | 0.620 | 2.13E-07 | 6.22E-05 |
| *MAMDC4* | 361.440 | 0.621 | 0.0001 | 0.0049 |
| *EBLN2* | 103.898 | 0.621 | 0.0031 | 0.0351 |
| *WBP1* | 410.367 | 0.624 | 1.46E-05 | 0.0012 |
| *NPIPB11* | 236.787 | 0.624 | 0.0011 | 0.0178 |
| *SCART1* | 149.862 | 0.625 | 0.0038 | 0.0392 |
| *CA3* | 107.449 | 0.626 | 0.0052 | 0.0473 |
| *SLC25A27* | 444.342 | 0.627 | 2.26E-05 | 0.0016 |
| *CTSK* | 257.020 | 0.627 | 0.0001 | 0.0052 |
| *CROCCP3* | 140.313 | 0.628 | 0.0005 | 0.0108 |
| *OVGP1* | 234.721 | 0.628 | 0.0003 | 0.0087 |
| *IFITM10* | 4210.078 | 0.631 | 6.34E-08 | 2.61E-05 |
| *ELFN2* | 124.812 | 0.632 | 0.0024 | 0.0302 |
| *KIFC2* | 1629.117 | 0.633 | 0.0043 | 0.0419 |
| *TCTEX1D1* | 424.539 | 0.634 | 4.79E-07 | 0.0001 |
| *ZNF273* | 448.383 | 0.634 | 8.13E-07 | 0.0002 |
| *CHRNA10* | 81.851 | 0.636 | 0.0054 | 0.0483 |
| *LOC100128288* | 89.215 | 0.637 | 0.0038 | 0.0390 |
| *SPAG8* | 995.953 | 0.638 | 1.34E-09 | 1.25E-06 |
| *SPACA6P* | 162.081 | 0.638 | 0.0022 | 0.0288 |
| *ATG16L2* | 1407.552 | 0.639 | 2.58E-06 | 0.0004 |
| *C5AR1* | 648.115 | 0.640 | 1.09E-08 | 6.79E-06 |
| *LINC00342* | 1335.795 | 0.640 | 2.61E-07 | 6.88E-05 |
| *PAQR6* | 385.323 | 0.642 | 2.36E-05 | 0.0016 |
| *TSC22D1-AS1* | 114.940 | 0.642 | 0.0009 | 0.0163 |
| *LOC100287497* | 140.750 | 0.644 | 0.0027 | 0.0323 |
| *LOC101928581* | 109.863 | 0.646 | 0.0045 | 0.0431 |
| *PRRT2* | 245.282 | 0.647 | 0.0038 | 0.0392 |
| *CCDC78* | 2006.187 | 0.648 | 5.37E-10 | 7.52E-07 |
| *LOC100506518* | 95.840 | 0.648 | 0.0014 | 0.0215 |
| *LOC101927910* | 147.046 | 0.650 | 0.0013 | 0.0199 |
| *FAM229A* | 139.530 | 0.651 | 0.0008 | 0.0144 |
| *LOC729732* | 179.707 | 0.652 | 0.0011 | 0.0176 |
| *HSD17B7P2* | 154.333 | 0.653 | 0.0001 | 0.0047 |
| *ICAM5* | 517.709 | 0.655 | 1.17E-09 | 1.25E-06 |
| *LOC100505771* | 240.641 | 0.655 | 1.08E-06 | 0.0002 |
| *RBM20* | 156.755 | 0.656 | 0.0006 | 0.0129 |
| *GOLGA8B* | 1188.801 | 0.656 | 0.0052 | 0.0472 |
| *RPL36A* | 198.965 | 0.659 | 7.11E-05 | 0.0032 |
| *IFNE* | 280.474 | 0.660 | 0.0002 | 0.0069 |
| *GOLGA2P5* | 910.903 | 0.661 | 1.88E-07 | 5.73E-05 |
| *LOC100132832* | 103.727 | 0.661 | 0.0032 | 0.0357 |
| *DLEC1* | 2274.563 | 0.663 | 6.71E-08 | 2.68E-05 |
| *AGAP6* | 627.215 | 0.665 | 5.39E-07 | 0.0001 |
| *PSD* | 113.579 | 0.666 | 0.0021 | 0.0279 |
| *DZIP1L* | 735.716 | 0.667 | 6.93E-09 | 4.85E-06 |
| *C9orf117* | 1235.021 | 0.668 | 1.27E-06 | 0.0002 |
| *LOC100507520* | 971.490 | 0.668 | 1.36E-06 | 0.0002 |
| *CGB7* | 127.940 | 0.671 | 0.0002 | 0.0070 |
| *SNORD74* | 81.182 | 0.672 | 0.0047 | 0.0444 |
| *NPIPB3* | 740.279 | 0.676 | 1.46E-05 | 0.0012 |
| *GOLGA8A* | 3124.537 | 0.676 | 0.0039 | 0.0396 |
| *CATSPER2* | 161.906 | 0.682 | 0.0006 | 0.0118 |
| *AGAP9* | 516.100 | 0.684 | 3.65E-05 | 0.0021 |
| *C2orf73* | 152.936 | 0.686 | 0.0007 | 0.0131 |
| *LOC613037* | 279.582 | 0.686 | 0.0004 | 0.0105 |
| *MSTO2P* | 127.991 | 0.686 | 0.0005 | 0.0106 |
| *C19orf71* | 142.349 | 0.687 | 0.0010 | 0.0170 |
| *LINC00672* | 172.794 | 0.690 | 0.0002 | 0.0060 |
| *LOC101929670* | 72.975 | 0.691 | 0.0054 | 0.0483 |
| *AMY2B* | 312.140 | 0.691 | 3.03E-05 | 0.0019 |
| *CAPN8* | 510.642 | 0.691 | 1.61E-05 | 0.0013 |
| *LINC00240* | 106.761 | 0.692 | 0.0009 | 0.0161 |
| *DNAH1* | 1246.124 | 0.696 | 3.84E-09 | 3.12E-06 |
| *TMEM178A* | 170.996 | 0.697 | 0.0003 | 0.0087 |
| *DNAAF1* | 1961.888 | 0.697 | 5.13E-08 | 2.19E-05 |
| *CDHR3* | 2226.272 | 0.697 | 4.40E-11 | 1.23E-07 |
| *WASH7P* | 122.318 | 0.697 | 0.0002 | 0.0070 |
| *INE1* | 109.605 | 0.699 | 0.0029 | 0.0340 |
| *MAPK15* | 3137.218 | 0.701 | 2.56E-09 | 2.24E-06 |
| *LINC01004* | 500.729 | 0.707 | 6.46E-06 | 0.0007 |
| *SSPO* | 72.072 | 0.713 | 0.0056 | 0.0497 |
| *KIAA0319* | 74.052 | 0.714 | 0.0043 | 0.0419 |
| *PSMD6-AS2* | 86.365 | 0.716 | 0.0009 | 0.0158 |
| *LOC101928524* | 279.612 | 0.723 | 2.42E-05 | 0.0016 |
| *MC1R* | 312.236 | 0.726 | 1.47E-05 | 0.0012 |
| *SLC23A3* | 80.582 | 0.726 | 0.0018 | 0.0251 |
| *KNDC1* | 224.100 | 0.727 | 0.0001 | 0.0047 |
| *LOC100288152* | 2254.528 | 0.727 | 0.0023 | 0.0295 |
| *RPL32P3* | 371.536 | 0.728 | 1.07E-08 | 6.79E-06 |
| *NEAT1* | 32507.585 | 0.728 | 3.48E-12 | 1.22E-08 |
| *SNORA70* | 125.760 | 0.731 | 0.0001 | 0.0049 |
| *ZNF300P1* | 117.723 | 0.733 | 0.0022 | 0.0288 |
| *SCNN1D* | 180.923 | 0.733 | 0.0014 | 0.0210 |
| *LOC100652768* | 170.296 | 0.735 | 8.30E-05 | 0.0036 |
| *LOC644794* | 325.252 | 0.735 | 4.50E-05 | 0.0024 |
| *LOC101927119* | 78.486 | 0.735 | 0.0034 | 0.0370 |
| *LOC100131564* | 609.429 | 0.736 | 3.91E-07 | 9.96E-05 |
| *NPIPB4* | 317.054 | 0.737 | 0.0002 | 0.0072 |
| *LOC729737* | 359.213 | 0.739 | 5.52E-06 | 0.0007 |
| *LOC102723566* | 116.689 | 0.741 | 0.0005 | 0.0109 |
| *RP5-1057J7.6* | 296.763 | 0.743 | 7.99E-06 | 0.0008 |
| *SGK494* | 228.308 | 0.745 | 2.51E-05 | 0.0017 |
| *N4BP2L2-IT2* | 104.441 | 0.746 | 0.0034 | 0.0372 |
| *USP32P2* | 101.743 | 0.746 | 0.0043 | 0.0423 |
| *LOC728763* | 3263.752 | 0.749 | 3.02E-10 | 4.70E-07 |
| *C21orf15* | 96.250 | 0.750 | 0.0028 | 0.0330 |
| *SLC22A20* | 128.234 | 0.751 | 9.15E-05 | 0.0039 |
| *COLQ* | 148.138 | 0.751 | 0.0005 | 0.0110 |
| *STAC3* | 111.928 | 0.755 | 0.0025 | 0.0306 |
| *DNHD1* | 336.968 | 0.758 | 1.92E-06 | 0.0003 |
| *FAM227A* | 1037.900 | 0.758 | 4.02E-09 | 3.12E-06 |
| *KLRAP1* | 122.073 | 0.758 | 0.0003 | 0.0082 |
| *ADM5* | 85.640 | 0.760 | 0.0013 | 0.0203 |
| *LOC284581* | 189.343 | 0.762 | 0.0006 | 0.0125 |
| *GNRH1* | 108.362 | 0.763 | 0.0006 | 0.0130 |
| *LOC100507424* | 108.137 | 0.769 | 0.0040 | 0.0404 |
| *LINC01187* | 91.696 | 0.770 | 0.0020 | 0.0272 |
| *MYOT* | 83.365 | 0.774 | 0.0009 | 0.0160 |
| *LCAT* | 276.275 | 0.777 | 1.81E-05 | 0.0014 |
| *C1orf228* | 314.546 | 0.777 | 2.33E-07 | 6.42E-05 |
| *NPFF* | 117.644 | 0.779 | 0.0002 | 0.0072 |
| *MIR205* | 54.197 | 0.780 | 0.0039 | 0.0396 |
| *SCXB* | 253.677 | 0.786 | 3.86E-06 | 0.0005 |
| *YJEFN3* | 157.021 | 0.790 | 0.0002 | 0.0056 |
| *CDHR4* | 1738.138 | 0.791 | 8.83E-10 | 1.03E-06 |
| *C6orf163* | 59.254 | 0.797 | 0.0028 | 0.0334 |
| *ATP2A1* | 60.277 | 0.798 | 0.0045 | 0.0430 |
| *FAM95C* | 421.376 | 0.806 | 1.32E-05 | 0.0012 |
| *LOC101928722* | 209.422 | 0.807 | 7.06E-06 | 0.0007 |
| *DYX1C1* | 124.991 | 0.807 | 6.74E-05 | 0.0031 |
| *AVIL* | 205.761 | 0.810 | 5.34E-05 | 0.0027 |
| *KCNQ1OT1* | 339.242 | 0.816 | 5.17E-08 | 2.19E-05 |
| *CCDC37* | 846.560 | 0.817 | 2.60E-10 | 4.54E-07 |
| *PRSS53* | 98.061 | 0.822 | 7.52E-05 | 0.0033 |
| *LINC00893* | 541.424 | 0.824 | 1.03E-06 | 0.0002 |
| *SERPINI2* | 167.461 | 0.824 | 8.37E-06 | 0.0008 |
| *VWA3A* | 1342.541 | 0.826 | 8.29E-10 | 1.03E-06 |
| *CCDC17* | 2893.525 | 0.833 | 9.06E-20 | 1.27E-15 |
| *EGLN3* | 134.152 | 0.836 | 0.0030 | 0.0348 |
| *SAP25* | 401.810 | 0.842 | 1.26E-08 | 7.36E-06 |
| *LOC100506124* | 88.730 | 0.844 | 0.0010 | 0.0164 |
| *SLC4A5* | 57.162 | 0.844 | 0.0014 | 0.0210 |
| *MYCBPAP* | 411.235 | 0.847 | 2.95E-08 | 1.43E-05 |
| *LINC00926* | 79.145 | 0.848 | 0.0023 | 0.0292 |
| *MIRLET7BHG* | 505.684 | 0.851 | 4.19E-07 | 1.05E-04 |
| *AGER* | 423.515 | 0.855 | 4.51E-08 | 2.04E-05 |
| *ITGB7* | 95.082 | 0.861 | 6.81E-05 | 0.0031 |
| *LOC100288175* | 70.844 | 0.867 | 0.0007 | 0.0131 |
| *INHA* | 62.576 | 0.868 | 0.0021 | 0.0278 |
| *LOC102723500* | 122.372 | 0.878 | 1.92E-05 | 0.0014 |
| *ASMTL-AS1* | 602.343 | 0.880 | 0.0035 | 0.0374 |
| *TMEM190* | 522.755 | 0.886 | 1.33E-09 | 1.25E-06 |
| *BEST4* | 191.203 | 0.897 | 0.0003 | 0.0088 |
| *LOC101926935* | 48.170 | 0.908 | 0.0023 | 0.0292 |
| *LAT* | 155.137 | 0.919 | 6.46E-06 | 0.0007 |
| *TNFSF14* | 879.474 | 0.926 | 3.78E-17 | 2.64E-13 |
| *DOC2A* | 1044.181 | 0.927 | 0.0002 | 0.0056 |
| *LOC101928055* | 134.936 | 0.945 | 5.43E-06 | 0.0007 |
| *AMH* | 91.804 | 0.950 | 5.31E-05 | 0.0026 |
| *ITIH4* | 134.773 | 0.959 | 2.08E-05 | 0.0015 |
| *LOC101928615* | 72.169 | 0.979 | 0.0009 | 0.0158 |
| *KIAA1875* | 161.444 | 0.993 | 6.15E-06 | 0.0007 |
| *MIR1914* | 64.259 | 0.995 | 0.0007 | 0.0133 |
| *LINC00894* | 133.783 | 0.998 | 8.24E-07 | 0.0002 |
| *LRRC36* | 54.977 | 0.999 | 0.0005 | 0.0116 |
| *CILP* | 103.814 | 1.002 | 3.02E-05 | 0.0019 |
| *HPX* | 82.798 | 1.008 | 0.0010 | 0.0169 |
| *LINC00954* | 39.490 | 1.011 | 0.0029 | 0.0337 |
| *MIR149* | 46.994 | 1.023 | 0.0019 | 0.0261 |
| *POU5F1* | 89.476 | 1.049 | 0.0003 | 0.0080 |
| *LOC400891* | 407.769 | 1.064 | 7.97E-11 | 1.59E-07 |
| *OVCH2* | 39.207 | 1.105 | 0.0013 | 0.0204 |
| *MYH3* | 69.048 | 1.120 | 1.34E-05 | 0.0012 |
| *C1QTNF8* | 47.197 | 1.143 | 0.0035 | 0.0374 |
| *LOC101929379* | 49.943 | 1.178 | 8.50E-05 | 0.0036 |
| *ASB14* | 53.703 | 1.196 | 7.56E-05 | 0.0033 |
| *TMEM52* | 90.128 | 1.240 | 1.63E-06 | 0.0003 |
| *LIMS2* | 52.830 | 1.260 | 0.0005 | 0.0110 |
| *SULT1A2* | 36.914 | 1.332 | 5.57E-05 | 0.0027 |
| *FER1L5* | 54.604 | 1.437 | 3.45E-06 | 0.0005 |
| *COL11A2* | 37.514 | 1.467 | 9.68E-06 | 0.0009 |
| *TNNI3* | 116.641 | 1.498 | 7.11E-11 | 1.59E-07 |
| *LOC100996273* | 67.920 | 1.588 | 1.68E-06 | 0.0003 |
